# The causal relationship between gut microbiota and leukemia: a two-sample Mendelian randomization study

**DOI:** 10.3389/fmicb.2023.1293333

**Published:** 2023-11-22

**Authors:** Guanjun Chen, Zheshu Kuang, Fan Li, Jianchang Li

**Affiliations:** ^1^Affiliated Hospital of Binzhou Medical University, Binzhou, China; ^2^Department of Gastroenterology, The First Hospital of Jilin University, Changchun, China

**Keywords:** gut microbiota, leukemia, mendelian randomization, acute lymphoblastic leukemia, acute myeloid leukemia, chronic lymphoblastic leukemia, chronic myeloid leukemia

## Abstract

**Background:**

The association between gut microbiota and leukemia has been established, but the causal relationship between the two remains unclear.

**Methods:**

A bidirectional two-sample Mendelian randomization (MR) was used to analyze the causal relationship between gut microbiota and leukemia. Microbiome data (*n* = 14,306) and leukemia (*n* = 1,145) data were both sourced from European populations. Single nucleotide polymorphisms (SNPs) were selected as instrumental variables based on several criteria. We employed various MR methods, such as the inverse variance weighted (IVW) method, to evaluate the causal effect between exposure and outcomes and conducted sensitivity analyses to validate the heterogeneity and pleiotropy of the instrumental variables.

**Results:**

5,742 qualified instrumental variables were included. In the primary MR results, a total of 10 gut microbial taxa were associated with leukemia risk. Genus Blautia and genus Lactococcus are risk factors for acute lymphoblastic leukemia [genus Blautia odds ratio (OR): 1.643, 95% confidence interval (CI): 1.592 ~ 1.695, Adjusted *p* < 0.001; genus Lactococcus OR: 2.152, 95% CI: 1.447 ~ 3.199, Adjusted *p* = 0.011]. Genus Rikenellaceae RC9 gut group, genus Anaerostipes, genus Slackia, and genus Lachnospiraceae ND3007 group are risk factors for acute myeloid leukemia [genus Rikenellaceae RC9 gut group OR: 1.964, 95% CI: 1.573 ~ 2.453, Adjusted *p* < 0.001; genus Anaerostipes OR: 2.515, 95% CI: 1.503 ~ 4.209, Adjusted *p* = 0.017; genus Slackia OR: 2.553, 95% CI: 1.481 ~ 4.401, Adjusted *p* = 0.022; genus Lachnospiraceae ND3007 group OR: 3.417, 95% CI: 1.960 ~ 5.959, Adjusted *p* = 0.001]. Genus Ruminococcaceae UCG011 and genus Ruminococcaceae UCG014 were risk factors for chronic myeloid leukemia (genus Ruminococcaceae UCG011 OR: 2.010, 95% CI: 1.363 ~ 2.963, Adjusted *p* = 0.044; genus Ruminococcaceae UCG014 OR: 3.101, 95% CI: 1.626 ~ 5.915, Adjusted *p* = 0.044). Genus Slackia was a protective factor for acute lymphoblastic leukemia (genus Slackia OR: 0.166, 95% CI: 0.062 ~ 0.443, Adjusted *p* = 0.017). Family Acidaminococcaceae was a protective factor for acute myeloid leukemia (family Acidaminococcaceae OR: 0.208, 95% CI: 0.120 ~ 0.361, Adjusted *p <* 0.001). Genus Desulfovibrio was a protective factor for chronic lymphoblastic leukemia (genus Desulfovibrio OR: 0.581, 95% CI: 0.440 ~ 0.768, Adjusted *p* = 0.020). Sensitivity analysis revealed no heterogeneity or pleiotropy between SNPs.

**Conclusion:**

This study revealed the causal relationship between the gut microbiota and leukemia, and identified potential pathogenic bacteria and probiotic taxa associated with the onset of leukemia. This research may aid in the early detection of various types of leukemia and offer a new direction for the prevention and treatment of leukemia.

## 1 Introduction

Leukemia is a malignant hematologic tumor, characterized by the metabolic dysregulation of leukemia cells and leukemia stem cells relative to non-cancerous stem cells (Nemkov et al., 2019). According to global cancer statistics, the incidence of Acute Lymphoblastic Leukemia (ALL) is approximately 1.7–4.6 individuals per 100,000 population, Acute Myeloid Leukemia (AML) is approximately 1.2–4.2 individuals per 100,000 population, Chronic Lymphocytic Leukemia (CLL) is approximately 2.0–5.0 individuals per 100,000 population, and Chronic Myeloid Leukemia (CML) is approximately 0.8–2.1 individuals per 100,000 population, with a continuing upward trend in recent years (Bray et al., 2018; Sung et al., 2021).

Currently, leukemia treatments primarily involve chemotherapy, radiation therapy, and hematopoietic stem cell transplantation. However, due to the complexity and heterogeneity of leukemia, therapeutic outcomes still have limitations. Hence, further research and exploration into the pathogenesis of leukemia and new therapeutic strategies are essential.

While the etiology and pathogenesis of leukemia are not fully understood, factors like immune dysfunction, genetic predispositions, and environmental elements play significant roles in its onset and progression. Moreover, increasing evidence suggests that alterations in the composition and function of gut microbiota are closely related to the onset, treatment, and prognosis of leukemia (Ma et al., 2021; Wang et al., 2022; Zhou et al., 2022). The gut microbiota refers to the microbial community living within the human gastrointestinal tract. Its symbiotic relationship with the host is intricate and is closely related to human health (Valdes et al., 2018).

In many leukemia studies, gut microbial dysbiosis has been observed. For instance, Bai L and colleagues found that ALL induces structural changes in the gut microbial community. Antibiotics significantly reduced α-diversity, but β-diversity remained unchanged. Bacteroidales and Enterococcaceae can be considered as biological markers for ALL (Bai et al., 2017). A case–control study reported that the diversity of the gut microbiota (Anaerostipes, Coprococcus, Roseburia, and Ruminococcus2) in children and adolescents with ALL is significantly lower than that in the control group (Rajagopala et al., 2016). Rashidi A and colleagues discovered that in AML patients, gut microbiota like acteroides, Alistipes, and Faecalibacterium are disrupted during induction chemotherapy. This disruption is persistent, and normal levels cannot be restored after chemotherapy completion (Rashidi et al., 2022). However, most studies are designed as case–control studies, making it challenging to determine exposure time and outcomes. Furthermore, in observational studies, the association between gut microbiota and leukemia can be easily confounded by factors such as age, environment, dietary patterns, and lifestyle (Rinninella et al., 2019). Thus, whether there is a causal relationship between gut microbiota and leukemia remains uncertain.

Mendelian randomization (MR) as a natural randomization approach is increasingly utilized to integrate summary data from genome-wide association studies (GWAS) (Greenland, 2018). It employs genetic variations associated with the exposure as proxies for the exposure to assess the association between exposure and outcome, and is widely adopted in researching the causal relationships of disease etiology. Additionally, MR can effectively eliminate potential biases and confounders (such as immune dysfunction, genetic factors, environmental factors, etc.), ensuring the reliability and validity of the experimental results (Davey Smith and Hemani, 2014). In this study, we employed a two-sample MR to analyze whether there’s a causal relationship between the composition of gut microbiota and the risk of leukemia.

## 2 Materials and methods

### 2.1 Data sources

This study utilizes a two-sample MR to explore the causal relationship between different types of gut microbiota and the onset of leukemia, with data sourced from publicly available GWAS summary statistics.

We obtained the GWAS summary data related to individual gut microbiota from the MiBioGen global consortium database. To ensure minimal heterogeneity, we selected only 150 datasets of genus and species, with an average of 5,376,402 available SNPs. Participants were from 24 countries, including the United States, Canada, and Germany, with a total sample size of 18,340 (Kurilshikov et al., 2021).^1^

The study acquired the 16S rRNA gene sequencing profiles and genotyping data of these participants. We incorporated the data from European ancestry participants of this study as exposure in the MR analysis.

The FinnGen project in the Finnish biobank aims to collect and analyze genomic and health data of 500,000 participants (Kurki et al., 2023).^2^ From FINNGEN, we obtained GWAS datasets of various leukemias, with each dataset containing nearly 287,136 individuals, among which 1,145 were leukemia patients. The dataset information used for the MR analysis is shown in Table 1, Supplementary Tables S1, S8 (Kurilshikov et al., 2021). No additional ethical approval or consent to participate was required because we used published studies and public summary statistics.

**Table 1:** Dataset information.

Datasets	Authors	Year	Population	Gender	ICD-10 coding	sample size	NSNP	Number of individuals			Median age at first event		
								All	Female	Male	All	Female	Male
Acute lymphocytic leukaemia	FinnGen	2023	European	Males and females	C910	287,320	20,167,370	184	102	82	19.82	16.49	23.97
Acute myeloid leukaemia	FinnGen	2023	European	Males and females	C920	287,367	20,167,370	231	88	143	61.52	55.02	65.53
Chronic lymphocytic leukaemia	FinnGen	2023	European	Males and females	C911	287,757	20,167,370	624	193	431	67.41	66.63	67.75
Chronic myeloid leukaemia	FinnGen	2023	European	Males and females	C921	287,242	20,167,370	106	51	55	55.51	54.58	56.37
Abundance of 150 Gut microbiota	Kurilshikov A	2021	European	Males and females	–	14,306	5,331,372(Ave.)	–	–	–	–	–	–

**Figure 1:**
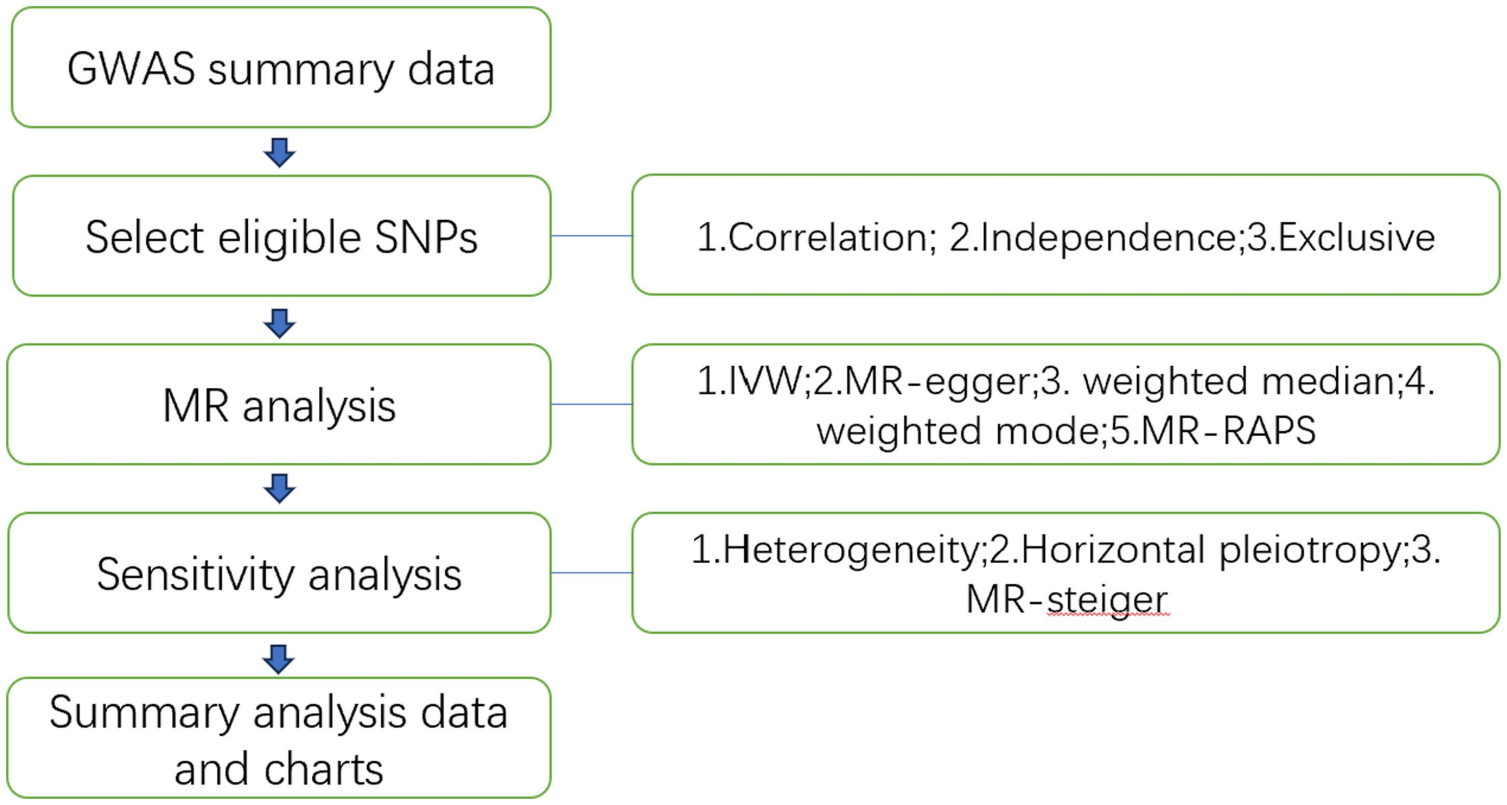
Study flowchart.

The study flowchart is illustrated in Figure 1. In essence, the gut microbiota is the exposure, and leukemia is the outcome. The leukemia diagnoses conform to the International Disease Classifications for ALL, AML, CLL, and CML, with their respective ICD10 codes being C910, C920, C911, and C921. Population stratification is a common source of bias in MR studies. To minimize this stratification, samples included in this MR study are exclusively from European populations, thereby reducing bias arising from population stratification (Larsson et al., 2019).

### 2.2 Basic assumptions of MR and instrumental variable (IV) selection

MR analysis employs Instrumental Variables (IVs) to estimate the relationship between exposure (gut microbiota) and outcomes (ALL, AML, CLL, CML), examining whether gut microbiota promotes or inhibits the onset of leukemia.

**Figure 2:**
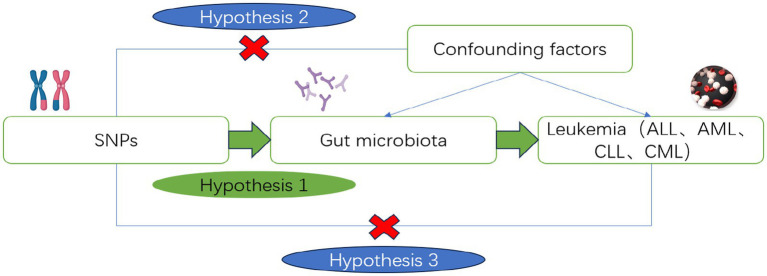
Directed acyclic diagram of the MR analysis principle. Created with MedPeer (www.medpeer.cn).

This study utilizes Single Nucleotide Polymorphisms (SNPs) as IVs. The instrumental variables used in MR analysis must meet the following basic assumptions: Assumption 1 (Relevance): The instrumental variable is significantly associated with the risk factor; Assumption 2 (Independence): The instrumental variable is not associated with any confounders; Assumption 3 (Exclusivity): The instrumental variable influences the outcome only indirectly through the risk factor. Using instrumental variables (SNPs), risk factors (gut microbiota), and outcomes (ALL, AML, CLL, CML), we constructed a Directed Acyclic Graph (DAG, Figure 2) to elucidate the aforementioned assumptions.

We applied a series of SNP selection criteria to the acquired GWAS summary data to ensure they met the three aforementioned assumptions:

Assumption 1: We selected SNPs associated with risk factors at *p*-value <1 × 10^−5^. Considering the limited number of SNPs, we slightly adjusted the threshold based on the genome-wide significance level (5 × 10^−8^) to meet the requirements for further analysis (Gou et al., 2023; Yue et al., 2023). We calculated the proportion of variance in exposure explained by SNP using the formula r2 = 2 * eaf * (1 − eaf) * beta^2 (Codd et al., 2013). To ensure the instrumental variables are independent genetic SNPs, we applied parameters r2 = 0.001 and physical distance = 10,000 base pairs to eliminate SNPs with linkage disequilibrium (LD). Using SNPs associated with risk factors, we extracted associated data from the outcome summary data and performed data matching. In this study, we removed ambiguous SNPs and palindromic SNPs during the matching process. Given the inevitable missing data in the outcome database and the spatiotemporal consistency among high-LD SNPs, we substituted missing SNPs in the outcome data with high-LD proxy SNPs, enhancing the model’s ability to interpret the true causal relationship. The criteria for selecting high linkage disequilibrium SNPs were r2 > 0.8 and window<10,000 kb. We calculated the F-statistic between SNP and exposure using the formula F = beta^2/se^2 to measure its strength as IVs (Burgess and Thompson, 2011). The F-statistic, used to gauge the strength of the instrumental variable in explaining the risk factor, considers SNPs with an F-statistic < 10 as weak instruments (Burgess et al., 2017), and these are excluded. The aforementioned filtering ensures a strong correlation between the SNP and the risk factors.

Assumption 2: We utilized phenoscanner to test potential associations of each SNP with confounders (Staley et al., 2016). SNPs that may violate the independence assumption need to be removed, ensuring the selected SNPs are not associated with any potential confounders. Mendelian Randomization Pleiotropy Residual Sum and Outlier (MR-PRESSO) analysis can detect and identify outliers and SNPs with pleiotropy (Verbanck et al., 2018), which are then excluded.

Assumption 3: Incorrect SNP selection might lead to outcomes directly associated with SNPs, eventually resulting in erroneous conclusions about reverse causal relationships. Therefore, we employed MR-steiger to test the causal estimation direction of SNPs (Hemani et al., 2017). SNPs with incorrect directions are excluded. Finally, based on the Bonferroni correction (*p* < 0.05/*n*, *n*: the number of remaining SNPs), we removed SNPs directly associated with the outcome. By following the above methods, we ensured that SNPs indirectly influence the outcome only through risk factors.

In summary, after the stringent filtering described above, the remaining SNPs were considered valid instrumental variables.

### 2.3 MR analysis study design

In this study, we employed the Inverse Variance Weighted (IVW) method, MR-Egger, Weighted Median, Weighted Mode and MR-RAPS to infer whether there’s a causal relationship between human gut microbiome composition and leukemia risk.

Essentially, IVW is a meta-analysis method, combining wald ratio estimates obtained from each SNP calculation using inverse variance weighting. This provides an overall estimate of the impact of the gut microbiota on leukemia risk, which is determined by dividing the effect estimate of the SNP result by the exposure effect estimate of the SNP (Burgess et al., 2013; Lee et al., 2016; Choi et al., 2019). When horizontal pleiotropy is absent, IVW can avoid the influence of confounders.

MR-Egger is similar to the IVW method, but the intercept term in the former can evaluate horizontal pleiotropy. Hence, MR-Egger regression was employed to assess whether the included SNPs have potential horizontal pleiotropy. The MR-Egger method is based on the NOME (No Measurement Error) assumption. Therefore, we calculated its I^2 value to quantify the extent to which MR-Egger violates the NOME assumption. When I^2 is less than 90%, the results require correction (Bowden et al., 2016). Moreover, given the lower accuracy and statistical power of the MR-Egger regression, we employed the MR-PRESSO method to detect outliers with pleiotropic bias and to correct for horizontal pleiotropy.

The weighted median and weighted mode methods can provide consistent causal effect estimates. Even if the majority of similar individual instrumental causal effect estimates come from valid instruments, the weighted median and weighted mode methods remain effective, even when other instruments do not meet the causal inference requirements of the MR method (Bowden et al., 2016; Hartwig et al., 2017). Compared to the MR-Egger method, the weighted median and weighted mode methods improve the accuracy of the results (Ooi et al., 2019).

Additionally, we also employed the MR-RAPS method, which directly uses a random-effects distribution to simulate the pleiotropy of genetic variants, making it more robust than traditional MR methods (Wu et al., 2020).

In the absence of heterogeneity and horizontal pleiotropy, the IVW MRE model serves as the primary analytical method. If heterogeneity is present, results from the IVW random-effects model and the weighted median method are discussed collectively. If horizontal pleiotropy exists among SNPs, the MR-Egger method is used as the primary analytical approach. Non-primary MR analytical methods serve as sensitivity analyses to assess the reliability of the aforementioned models. The final results of this study are considered statistically significant at a significance level of *p* < 0.05.

### 2.4 Sensitivity analysis

Sensitivity analysis serves as the assessment of uncertain factors in a model to reduce their impact on the results in subsequent operations. Therefore, in addition to using comprehensive MR-steiger analysis to ensure the overall direction of causal effects and various MR analyses to explore the causal relationship between human gut microbiota composition and leukemia risk, we also conducted heterogeneity tests, pleiotropy analyses, and other sensitivity analyses.

We employed Cochran’s Q test to quantify the heterogeneity among the selected SNPs. A *p*-value <0.05 for the Q statistic indicates the presence of heterogeneity. The intercept term of MR-Egger can assess horizontal pleiotropy. When the analysis results show significant horizontal pleiotropy, the causal estimation results of MR-Egger are preferred.

### 2.5 Statistical software and data visualization

All statistical analyses in this study were performed using R software (version 4.1.2) and employed the R package “TwoSampleMR” along with some basic R packages.

The study produced scatter plots for each SNP’s impact on risk factors and outcomes, as well as regression curves for causal estimates. Forest plots, scatter plots of MR analysis, and fitted curve diagrams were drawn based on the final causal estimation results. Additionally, we produced plots using the leave-one-out method to assess whether the MR conclusion is dependent on a particular SNP. If such an SNP exists in a significant MR conclusion, consideration should be given to removing that instrumental variable.

## 3 Results

### 3.1 Filtering qualified SNPs

To investigate the causal influence of gut microbiota on leukemia, we selected 7,291 significantly associated SNPs. Due to missing data for some SNPs in the result dataset, we removed 368 associated SNPs. The F-statistics for each SNP was >10, indicating that there were no weak instrumental variables. Subsequently, we eliminated 1,120 palindromic and ambiguous SNPs. Using Phenoscanner, we manually removed 20 SNPs related to confounding factors, such as viral infections (Bartenhagen et al., 2017), immune abnormalities (Rutella et al., 2022), chemical exposure (Beane Freeman et al., 2009), ionizing radiation exposure (Metz-Flamant et al., 2012), and alcohol consumption (Engen et al., 2015). The MR-PRESSO analysis identified and removed 19 SNPs with horizontal pleiotropy and outliers. The MR-steiger analysis did not identify SNPs with incorrect causal directions. 22 SNPs directly associated with the outcome were removed after Bonferroni correction.

After rigorous selection, a total of 5,742 SNPs were included as qualified IVs for subsequent MR analysis. Detailed information about the selected instrumental variables is presented in Supplementary Tables S3–S5.

### 3.2 MR analysis results

**Figure 3:**
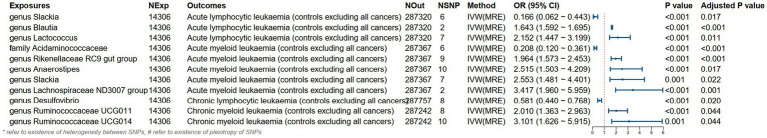
MR results and its forest plot.

**Figure 4:**
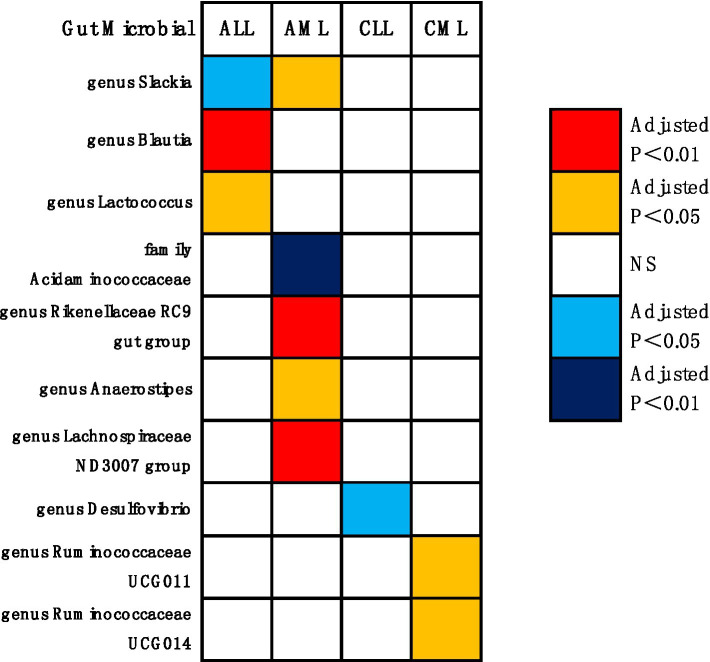
Causal effects of gut microbiota on leukemia (*p* < 0.05/0.01). Red indicates risk factors for leukemia, blue represents protective factors for leukemia, and white signifies non-causal factors for leukemia. NS, not significantly associated.

As shown in Figures 3, 4, the MR analysis results using the IVW method revealed 10 gut microbiota genera associated with the risk of leukemia.

Genus Blautia and genus Lactococcus were risk factors for ALL (genus Blautia OR: 1.643, 95% CI: 1.592 ~ 1.695, Adjusted *p* < 0.001; genus Lactococcus OR: 2.152, 95% CI: 1.447 ~ 3.199, Adjusted *p* = 0.011). Genus Rikenellaceae RC9 gut group, genus Anaerostipes, genus Slackia, and genus Lachnospiraceae ND3007 group were risk factors for AML (genus Rikenellaceae RC9 gut group OR: 1.964, 95% CI: 1.573 ~ 2.453, Adjusted *p* < 0.001; genus Anaerostipes OR: 2.515, 95% CI: 1.503 ~ 4.209, Adjusted *p* = 0.017; genus Slackia OR: 2.553, 95% CI: 1.481 ~ 4.401, Adjusted *p* = 0.022; genus Lachnospiraceae ND3007 group OR: 3.417, 95% CI: 1.960 ~ 5.959, Adjusted *p* = 0.001). Genus Ruminococcaceae UCG011 and genus Ruminococcaceae UCG014 were risk factors for CML (genus Ruminococcaceae UCG011 OR: 2.010, 95% CI: 1.363 ~ 2.963, Adjusted *p* = 0.044; genus Ruminococcaceae UCG014 OR: 3.101, 95% CI: 1.626 ~ 5.915, Adjusted *p* = 0.044). Genus Slackia was a protective factor for ALL (genus Slackia OR: 0.166, 95% CI: 0.062 ~ 0.443, Adjusted *p* = 0.017). Family Acidaminococcaceae was a protective factor for AML (family Acidaminococcaceae OR: 0.208, 95% CI: 0.120 ~ 0.361, Adjusted *p* < 0.001). Genus Desulfovibrio was a protective factor for CLL (genus Desulfovibrio OR: 0.581, 95% CI: 0.440 ~ 0.768, Adjusted *p* = 0.020).

Additionally, as indicated in Supplementary Figure S4, preliminary MR results suggest an association between leukemia occurrence and the gut microbiota such as genus Christensenellaceae R 7 group, family Family XIII, and family Family XI. However, these associations were no longer significant after FDR correction (Adjusted *p* > 0.05).

Supplementary Figure S1 presents the forest plot of the final causal estimation results. The scatter plot in Supplementary Figure S2 illustrates the causal effects of SNPs on the gut microbiota and leukemia. The leave-one-out analysis in Supplementary Figure S3 did not identify any SNPs that significantly influenced the outcome.

### 3.3 Sensitivity analysis results

In the sensitivity analysis, we conducted heterogeneity tests, horizontal pleiotropy analysis, and a comprehensive MR-steiger analysis, with the results presented in Table 2.

**Table 2:** Tests of MR-Steiger casual direction, MR-Egger I2, heterogeneity and pleiotropy.

Exposures	Outcomes	Q from IVW	Q from MR-Egger	Pval_Q from IVW	Pval_Q from MR-Egger	MR-Steiger	Pval of MR-Egger interception	I2 of MR-Egger
Genus Blautia	Acute lymphocytic leukaemia (controls excluding all cancers)	–	–	–	–	True	–	0.55
Genus Rikenellaceae RC9 gut group	Acute myeloid leukaemia (controls excluding all cancers)	1.36	0.97	0.99	1.00	True	0.55	0.95
Family Acidaminococcaceae	Acute myeloid leukaemia (controls excluding all cancers)	1.16	0.94	0.95	0.92	True	0.67	0.96
Genus Lachnospiraceae ND3007 group	Acute myeloid leukaemia (controls excluding all cancers)	–	–	–	–	True	–	0.00
Genus Lactococcus	Acute lymphocytic leukaemia (controls excluding all cancers)	1.50	1.50	0.96	0.91	True	0.95	0.89
Genus Slackia	Acute lymphocytic leukaemia (controls excluding all cancers)	4.46	4.24	0.49	0.37	True	0.67	0.98
Genus Anaerostipes	Acute myeloid leukaemia (controls excluding all cancers)	1.72	1.70	1.00	0.99	True	0.89	0.83
Genus Desulfovibrio	Chronic lymphocytic leukaemia (controls excluding all cancers)	1.66	1.66	0.98	0.95	True	0.99	0.93
Genus Slackia	Acute myeloid leukaemia (controls excluding all cancers)	2.43	2.41	0.88	0.79	True	0.89	0.92
Genus Ruminococcaceae UCG011	Chronic myeloid leukaemia (controls excluding all cancers)	1.51	1.51	0.98	0.96	True	0.98	0.92
Genus Ruminococcaceae UCG014	Chronic myeloid leukaemia (controls excluding all cancers)	2.07	1.99	0.99	0.98	True	0.78	0.90

MR studies with fewer than 3 SNPs cannot undergo Q-test and MR-Egger intercept analysis.

The heterogeneity test revealed no apparent heterogeneity among the SNP groups within the investigated gut microbiota variables. We calculated the intercept term of the MR-Egger regression and found no evidence of horizontal pleiotropy. Lastly, to ensure the overall causal effect direction of the MR analysis, we applied the MR-steiger model to validate the direction of the causal estimation. The results confirmed that the direction of causality in every MR analysis was correct.

The estimations of the association between the gut microbiota and leukemia using various MR methods are presented in Supplementary Table S2. Results for heterogeneity tests and horizontal pleiotropy analysis are shown in Supplementary Tables S6, S7, respectively. The power of each MR study can be found in Supplementary Table S2.

## 4 Discussion

The relationship between the gut microbiota and leukemia is a research area that has garnered significant attention. As mentioned in the background, several studies have indicated that the gut microbiota may play a role in the onset and progression of leukemia.

To the best of our knowledge, this study is among the first to systematically assess the causal relationship between the gut microbiota and various types of leukemia. Our MR research, utilizing a large-scale GWAS database, fills a gap in this research domain from a novel perspective.

Two-sample MR studies provide compelling evidence suggesting that specific gut microbes play a substantial role in the onset and progression of various leukemias, with metabolites from these microbiota playing a crucial role. Traditional observational studies, influenced by confounders like dietary habits and age, as well as reverse causality (Davies et al., 2018), may present biased findings. Therefore, utilizing SNPs as IVs in MR analysis to explore the causal relationship between gut microbiota and leukemia substantially mitigates these interferences, rendering the conclusions more convincing.

Traditional observational studies have reported associations between variations in gut microbiota and leukemia. A prospective clinical study involving 29 AML patients, 17 CML patients, and 33 healthy participants found an increased relative abundance of Actinobacteria, Acidobacteria, and Chloroflexi at the phylum level, while a decrease in Tenericutes. At the genus level, especially in AML patients, there was an increase in Streptococcus and a decrease in Megamonas, Lachnospiraceae NC2004 group, and Prevotella 9 (Yu et al., 2021). Another study, using a 16S rRNA quantitative array and bioinformatic analysis to examine fecal samples from participants, found significant bacterial variations in the fecal specimens of ALL patients compared to healthy controls. For instance, there was a notable enrichment of *Bacteroides clarus* in ALL patients (Song and Gyarmati, 2020). Moreover, leukemia treatments can alter the gut microbiota. For instance, antibiotic treatment that down-regulates the gut microbiota can restore the anti-leukemia efficacy of APO866 (ElMokh et al., 2022). Using B-cell receptor inhibitors (BCRi) as targeted therapy can up-regulate the Bacteroidia levels in CLL patients (Zuccaro et al., 2023). This also seems to underscore the association between the gut microbiota and leukemia.

The molecular mechanisms through which gut microbiota induce leukemia onset and progression have attracted significant research attention. Studies have shown that dysbiosis of the gut microbiota may lead to immune system abnormalities, thereby increasing the risk of leukemia onset (Rooks and Garrett, 2016). A case–control study found that gastrointestinal infections increase the risk of AML, potentially due to immune dysregulation caused by the imbalance in the gut microbiota brought about by the infections (Østgård et al., 2018).

Secondly, metabolites derived from the gut microbiota play a pivotal role in the occurrence and progression of leukemia in relation to the gut microbiota. The gut microbiota can break down dietary fiber, producing short-chain fatty acids (SCFAs) like acetate, propionate, and butyrate. These SCFAs play a crucial role in the health of intestinal cells and the regulation of the immune system (Ganapathy et al., 2013; Swanson et al., 2020). Gut microbes such as genus Slackia, genus Blautia, genus Anaerostipes, genus Rikenellaceae RC9 gut group, genus Lachnospiraceae ND3007 group, and genus Ruminococcaceae UCG014 can produce substantial amounts of butyrate through various metabolic pathways. Research has shown that SCFAs are particularly crucial in inducing T lymphocyte differentiation and development, thereby driving anti-pathogen immunity or immune tolerance based on the immune environment, indirectly promoting the onset of leukemia (Zhang et al., 2019). For instance, SCFAs can promote microbial antigen-specific Th1 cells to produce IL-10 via the GPR43 signaling pathway. Furthermore, SCFAs can accelerate the expression of B-lymphocyte-induced maturation protein 1 (Blimp-1) in Th1 cells by activating the STAT3 and mTOR pathways, thereby enhancing Th1 cell production of IL-10, resulting in immune suppression (Sun et al., 2018). Butyrate, via the GPR41 and GPR43 signaling pathways, accelerates the metabolism of antigen-activated CD8+ T cells, thereby enhancing their memory potential (Bachem et al., 2019).

Tet2 gene is a tumor-suppressor gene, and Tet2 gene mutation is one of the factors that can trigger AML and CML (Chiba, 2016). Meisel et al. (2018) found that mutations in the Tet2 gene disrupt the integrity of the intestinal barrier, leading to the entry of gut microbiota into the bloodstream or local lymph nodes. The body’s immune system produces the corresponding inflammatory cytokine IL-6, which subsequently stimulates the proliferation of leukemia cells. Additionally, studies have indicated that inflammatory stimuli weaken the integrity of the intestinal barrier. The direct contact of gut microbiota and their metabolites (e.g., SCFAs) with the intestinal epithelium leads to the overactivation of NF-KB in intestinal epithelial cells. This promotes the production of cytokines such as TNF, IL-1, and IL-6, thereby inducing and exacerbating leukemia (Gerloff et al., 2015; Tsilimigras et al., 2017; Zhang et al., 2017).

A study found that in an ALL mouse model, the proportion of bacteria with dietary flavonoid conversion functions, such as the genus Lactococcus, increased (Song and Gyarmati, 2020). Dietary bioflavonoids have been shown to induce mixed-lineage leukemia (MLL) gene cleavage by targeting topoisomerase II, and they might lead to pediatric leukemia (Strick et al., 2000).

The aforementioned findings are consistent with our experimental results, strongly confirming that the abnormal composition of the gut microbiota is related to the pathogenesis of leukemia.

Probiotics are a class of active microorganisms that, by reducing gut dysbiosis, promoting nutrient absorption, protecting the intestinal mucosal barrier, modulating immunity, or inhibiting intestinal inflammation, have a positive impact on gut health (Fredricks, 2019).

The genus Desulfovibrio is a sulfate-reducing bacterium (SRB), and its metabolic pathways help maintain the sulfur cycle in the gut environment. In previous studies, the genus Desulfovibrio has been regarded as a “double-edged sword,” being both beneficial (Chen et al., 2015; Zhao et al., 2016) and harmful (Singh et al., 2023). The family Acidaminococcaceae consists of bacteria that play a crucial role in amino acid metabolism in the gut. Its imbalance is associated with the onset and progression of various diseases, including oral infections (Luo et al., 2020) and inflammatory bowel diseases (Gevers et al., 2014). However, no current research explicitly indicates a direct relationship between the two and leukemia.

Our study revealed that genus Desulfovibrio and family Acidaminococcaceae serve as protective factors for CLL and AML, potentially acting as probiotics to reduce the risk of CLL and AML. However, previous studies have demonstrated differential effects of both on human health, necessitating further randomized controlled trials to validate these findings. Additionally, the potential mechanisms through which genus Desulfovibrio and family Acidaminococcaceae inhibit leukemia development require further investigation.

Interestingly, the genus Slackia acts as a risk factor for ALL while serving as a protective factor for AML in the development and progression of leukemia. One possible reason is that ALL is more prevalent in children and adolescents, while AML mainly occurs in adults; thus, there are variations in gut microbiota across different age groups. Furthermore, the etiology, pathogenesis, and immune phenotypes of the two diseases differ. As a result, the relationship between different diseases and the same gut microbiota can vary, sometimes even being completely opposite.

Implementing the MR method can elucidate causal relationships from exposure to outcome, unaffected by confounding factors and reverse causation, potentially providing more convincing evidence than observational studies.

The MR conducted in our study has several advantages: Firstly, this research represents the first attempt to infer the causal relationship between the gut microbiota and leukemia using comprehensive GWAS data, offering a novel approach to select candidate gut microbiota for subsequent functional studies. Secondly, it is based on publicly available large-scale GWAS summary statistics, thus offering an efficient option without additional experimental costs to extract reliable genetic information. Thirdly, our research is comprehensive in its investigation of leukemia, having conducted a holistic analysis of the four primary types of leukemia. This affords us the opportunity to evaluate common gut microbiota that have causal relationships with various leukemias.

However, certain limitations should be mentioned: Firstly, the gut microbiota GWAS is still in its nascent phase in terms of sample size, which offers insufficient information at the species or strain level. Secondly, further subgroup analyses are not feasible due to the absence of stratified summary data (e.g., gender and ethnicity) in the initial studies. Thirdly, our study lacks *in vivo* or *in vitro* models. Future research directions can build upon this to establish a direct link between gut microbiota and the origins of leukemia. Fourthly, the power of some aspects of this study is relatively low, which may increase the probability of Type II errors. Lastly, given that the majority of GWAS participants are of European ancestry, extrapolating the study results to other ethnic groups may be limited.

## 5 Conclusion

In summary, through two-sample MR analyses using publicly available GWAS summary data, we evaluated the causal relationship between the gut microbiota and leukemia, and identified potential pathogenic bacteria and probiotic taxa that influence the onset of leukemia. This research may aid in the early detection of various types of leukemia and offer a new direction for the prevention and treatment of leukemia.

## Data availability statement

The datasets presented in this study can be found in online repositories. The names of the repository/repositories and accession number(s) can be found in the article/Supplementary material.

## Author contributions

GC: Conceptualization, Investigation, Writing – original draft, Writing – review & editing. ZK: Formal analysis, Supervision, Writing – review & editing. FL: Data curation, Software, Writing – review & editing. JL: Supervision, Validation, Writing – review & editing.

## Footnotes

^1^ https://mibiogen.gcc.rug.nl/

^2^ https://r9.finngen.fi/
