# Supplementary material for: The causal relationship between gut microbiota and leukemia: a two-sample Mendelian randomization study
Source: Front Microbiol. 2023 Nov 22;14:1293333. doi: 10.3389/fmicb.2023.1293333 (PMC10703164; doi:10.3389/fmicb.2023.1293333)
Supplement: Supplementary file 3 [file Image_2.pdf]

Supplemental Figure 2. Scatter plots for the causal association between gut microbiota and leukemia.

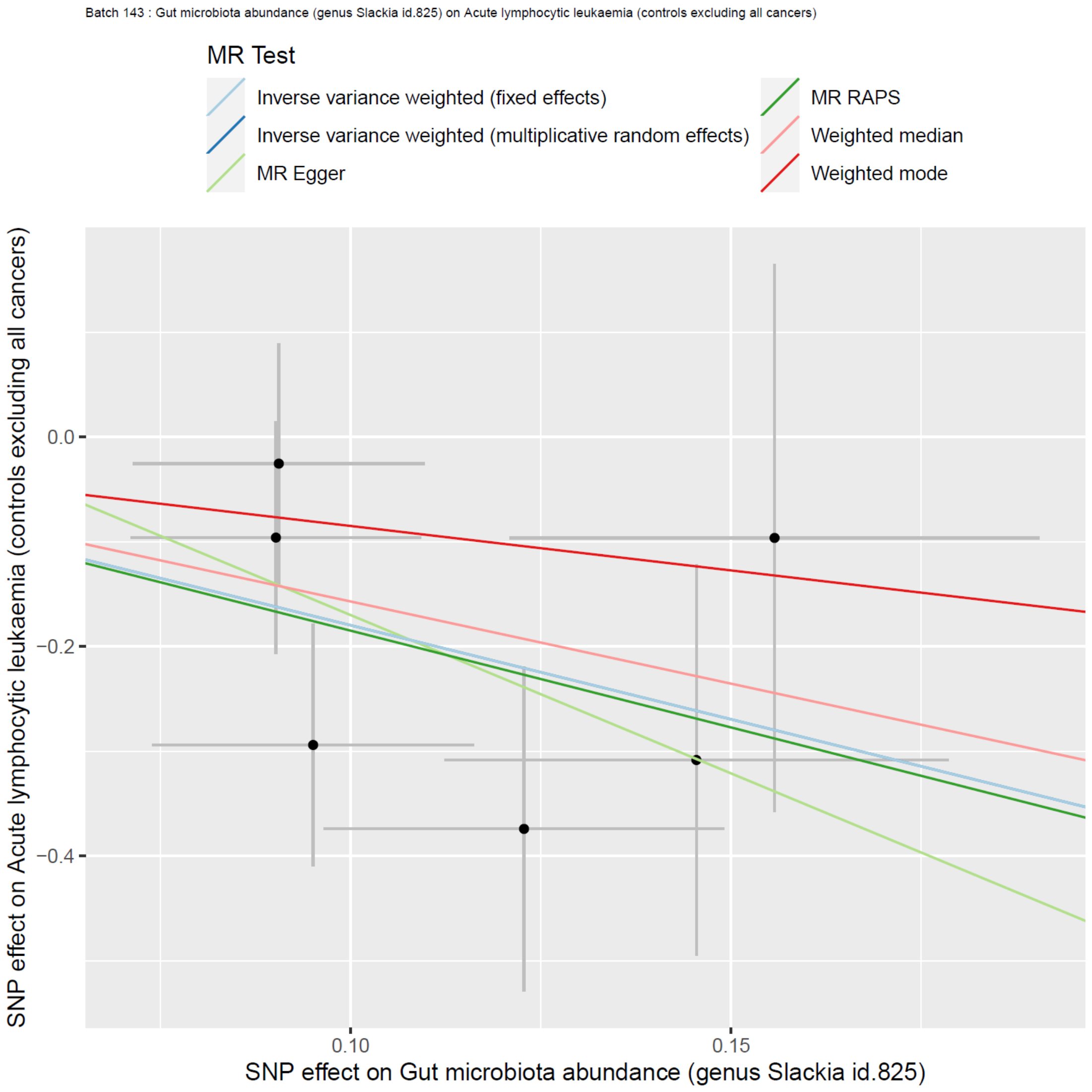

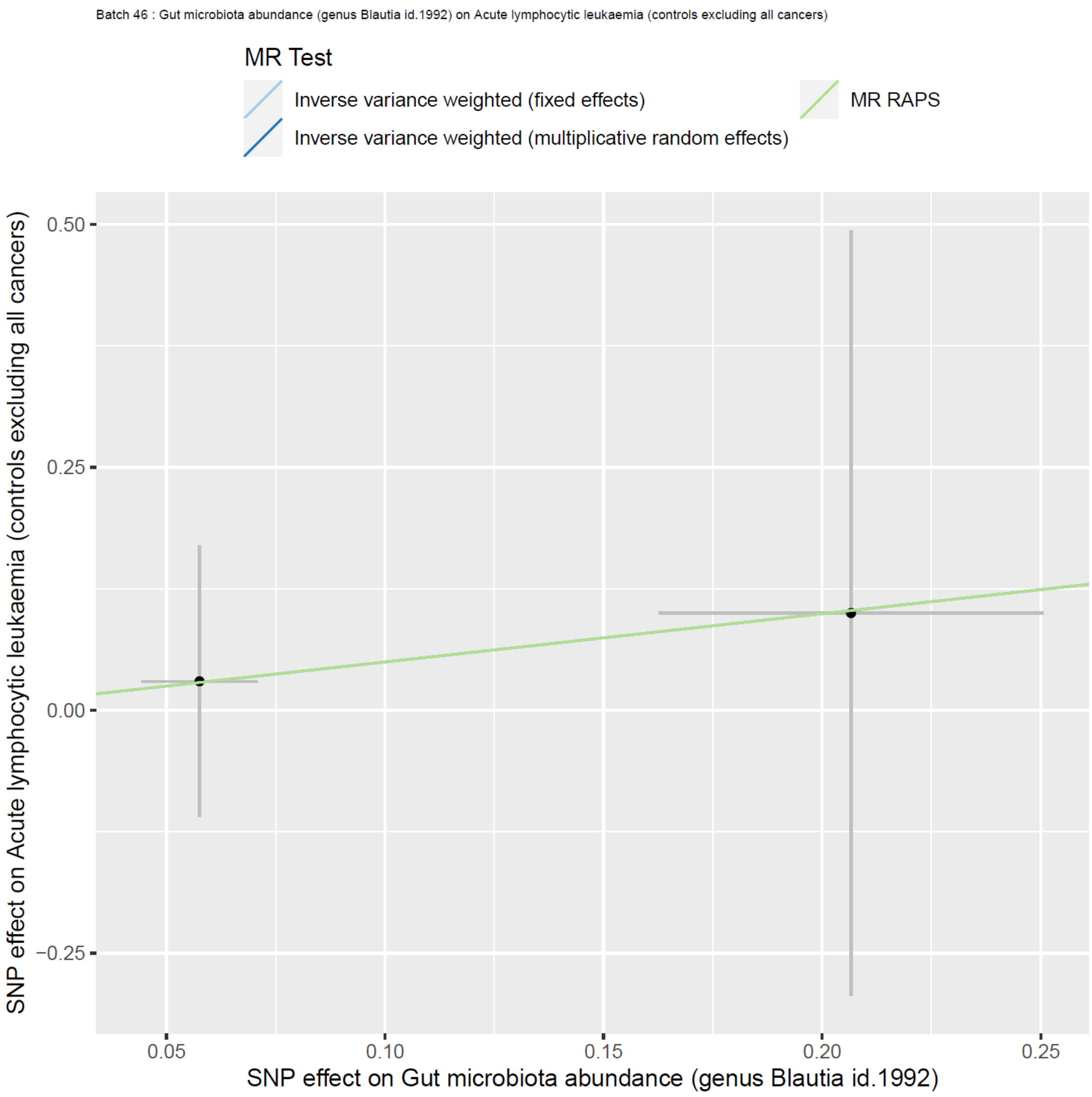

Batch 46 : Gut microbiota abundance (genus Blautia id.1992) on Acute lymphocytic leukaemia (controls excluding all cancers)
MR Test
Inverse variance weighted (fixed effects)
Inverse variance weighted (multiplicative random effects)
MR RAPS
0.50
0.25
0.00
−0.25
0.05
0.10
0.15
0.20
0.25
SNP effect on Acute lymphocytic leukaemia (controls excluding all cancers)
SNP effect on Gut microbiota abundance (genus Blautia id.1992)

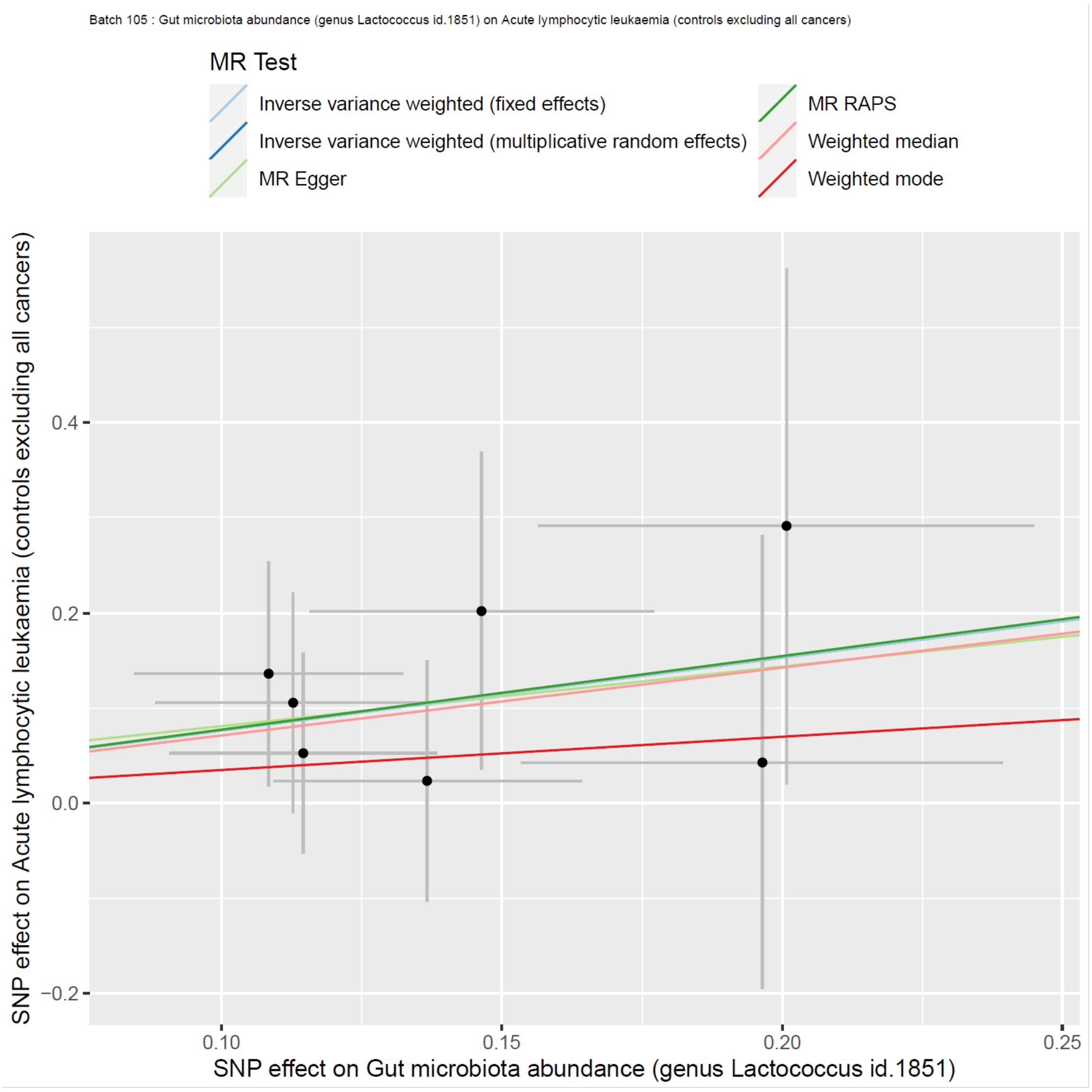
Batch 105 : Gut microbiota abundance (genus Lactococcus id.1851) on Acute lymphocytic leukaemia (controls excluding all cancers)
MR Test
Inverse variance weighted (fixed effects)
Inverse variance weighted (multiplicative random effects)
MR Egger
MR RAPS
Weighted median
Weighted mode
SNP effect on Acute lymphocytic leukaemia (controls excluding all cancers)
0.4
0.2
0.0
−0.2
0.10
0.15
0.20
0.25
SNP effect on Gut microbiota abundance (genus Lactococcus id.1851)

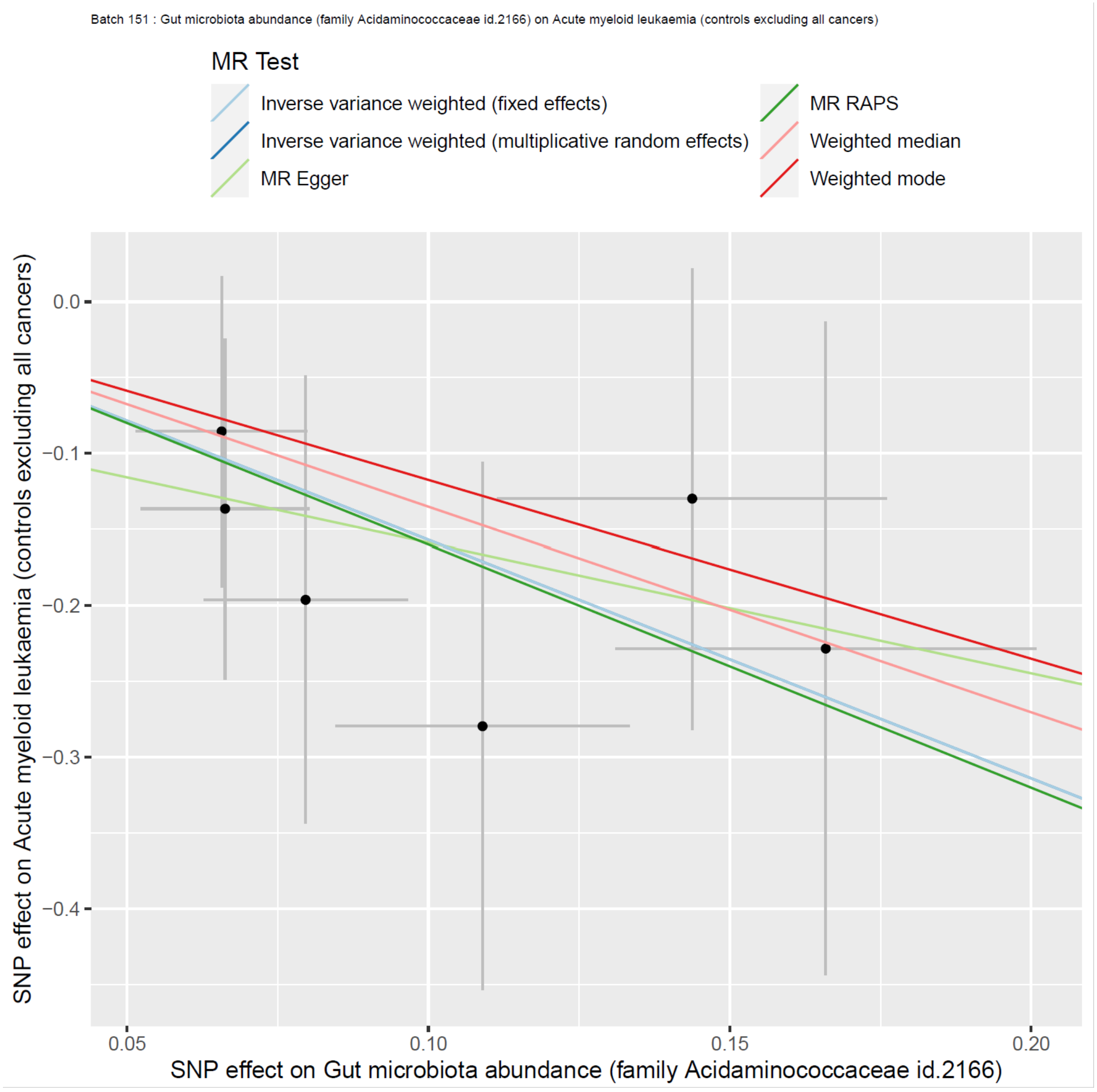

Batch 151 : Gut microbiota abundance (family Acidaminococcaceae id.2166) on Acute myeloid leukaemia (controls excluding all cancers)
MR Test
Inverse variance weighted (fixed effects)
Inverse variance weighted (multiplicative random effects)
MR Egger
MR RAPS
Weighted median
Weighted mode
SNP effect on Acute myeloid leukaemia (controls excluding all cancers)
0.0
−0.1
−0.2
−0.3
−0.4
0.05
0.10
0.15
0.20
SNP effect on Gut microbiota abundance (family Acidaminococcaceae id.2166)

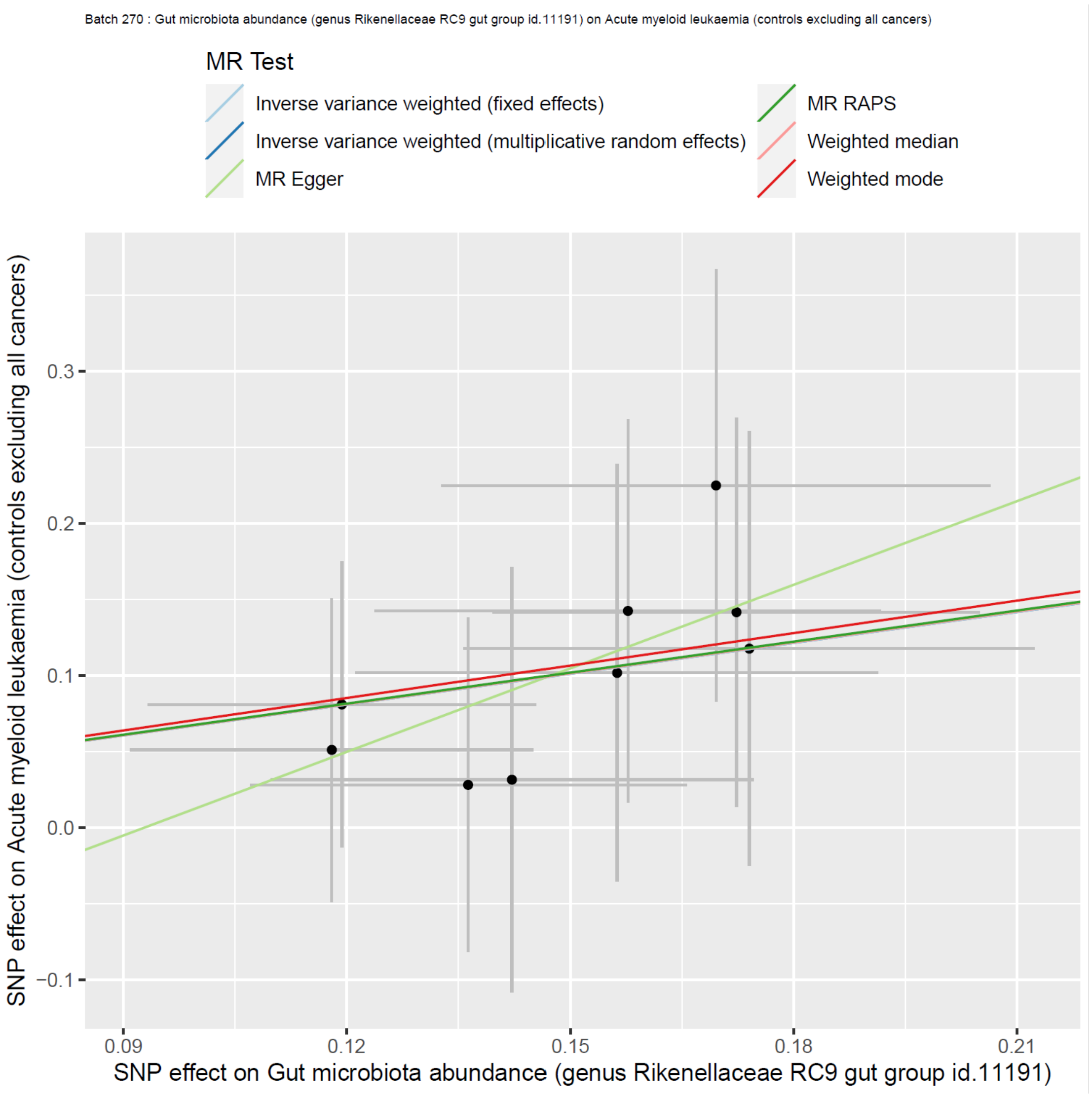
Batch 270 : Gut microbiota abundance (genus Rikenellaceae RC9 gut group id.11191) on Acute myeloid leukaemia (controls excluding all cancers)
MR Test
Inverse variance weighted (fixed effects)
Inverse variance weighted (multiplicative random effects)
MR Egger
MR RAPS
Weighted median
Weighted mode
SNP effect on Acute myeloid leukaemia (controls excluding all cancers)
0.3
0.2
0.1
0.0
−0.1
0.09
0.12
0.15
0.18
0.21
SNP effect on Gut microbiota abundance (genus Rikenellaceae RC9 gut group id.11191)

Batch 190 : Gut microbiota abundance (genus Anaerostipes id.1991) on Acute myeloid leukaemia (controls excluding all cancers)

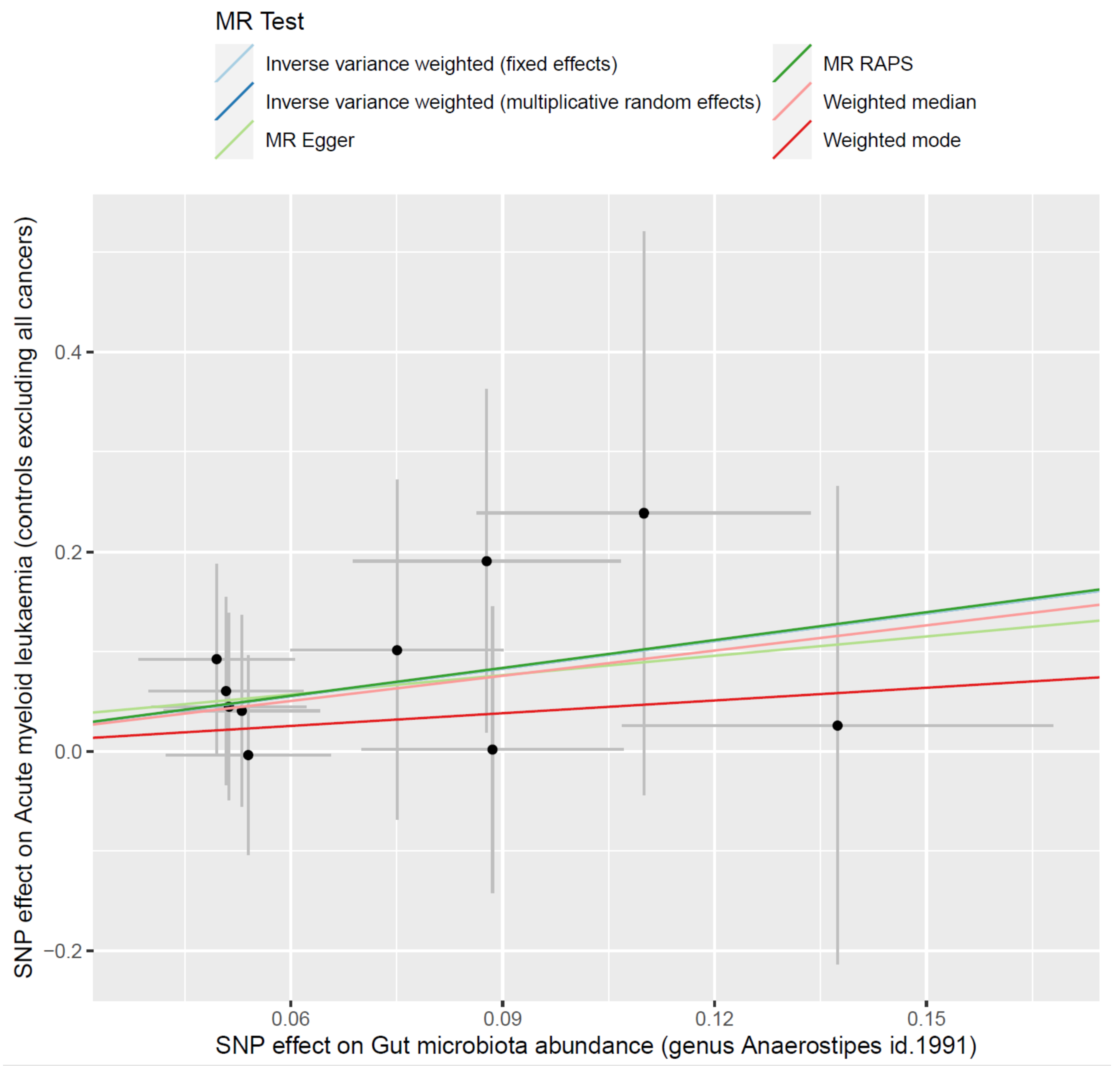

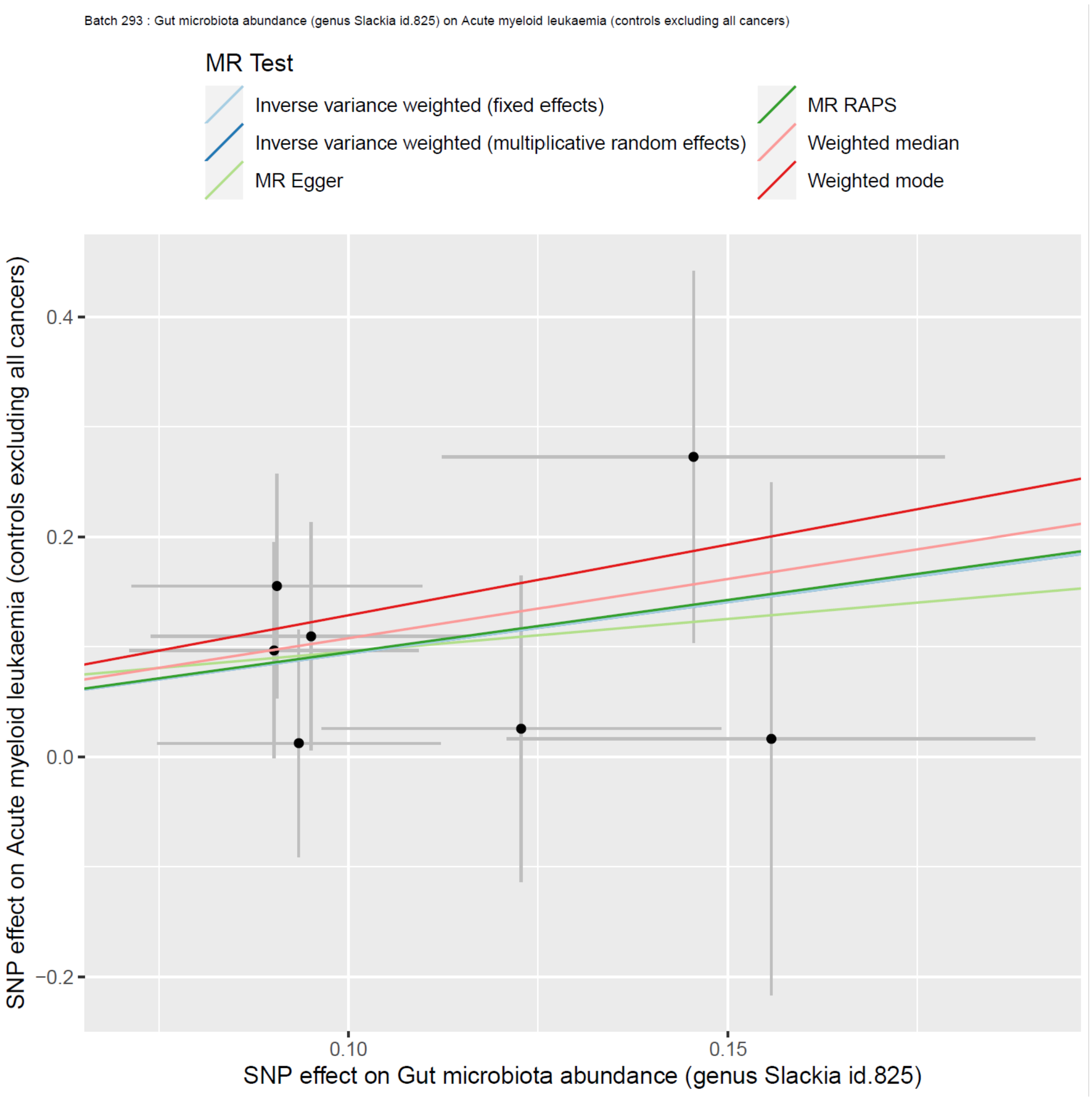
Batch 293 : Gut microbiota abundance (genus Slackia id.825) on Acute myeloid leukaemia (controls excluding all cancers)
MR Test
Inverse variance weighted (fixed effects)
Inverse variance weighted (multiplicative random effects)
MR Egger
MR RAPS
Weighted median
Weighted mode
SNP effect on Acute myeloid leukaemia (controls excluding all cancers)
0.4
0.2
0.0
−0.2
0.10
0.15
SNP effect on Gut microbiota abundance (genus Slackia id.825)

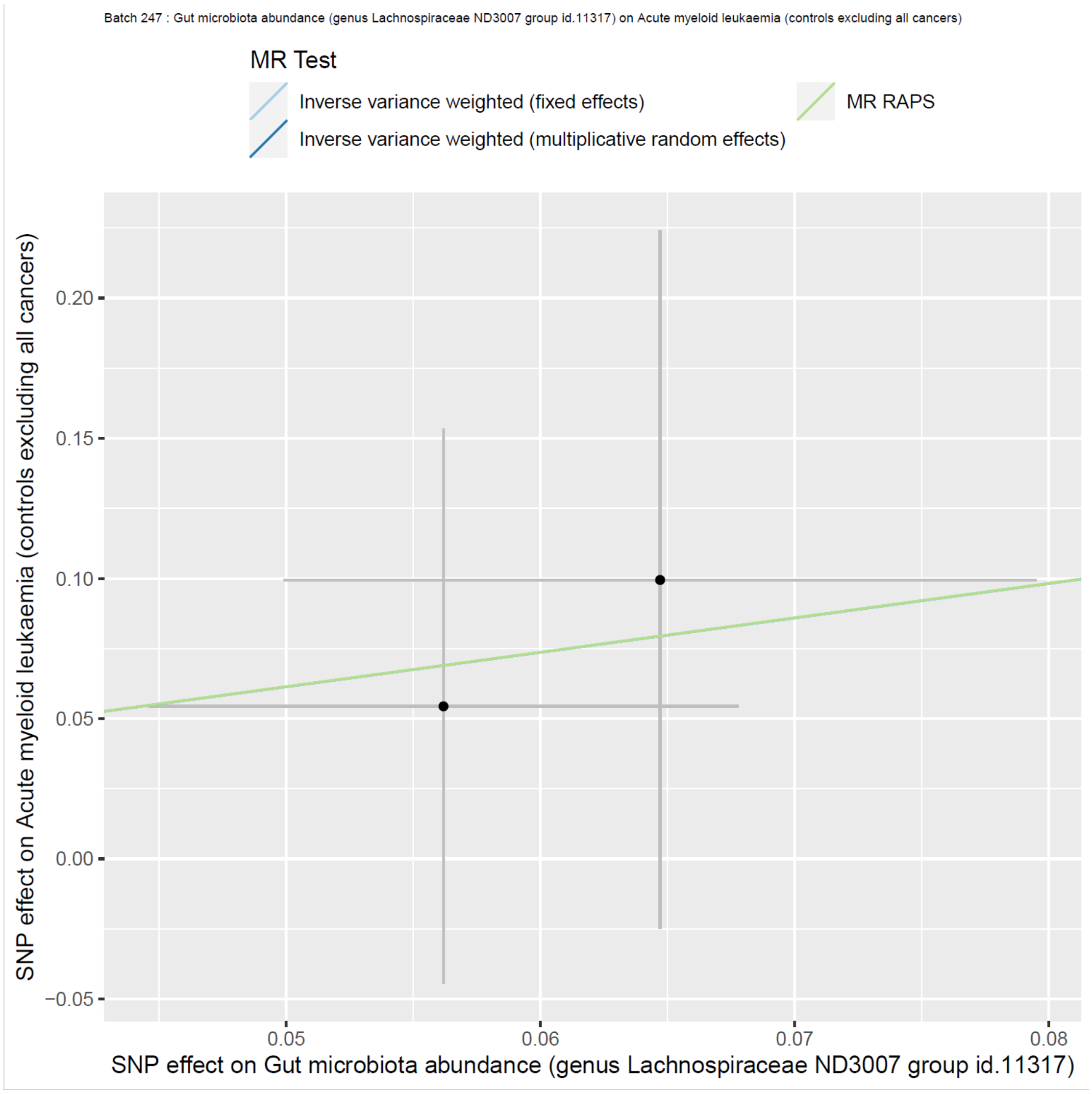

Batch 247 : Gut microbiota abundance (genus Lachnospiraceae ND3007 group id.11317) on Acute myeloid leukaemia (controls excluding all cancers)
MR Test
Inverse variance weighted (fixed effects)
Inverse variance weighted (multiplicative random effects)
MR RAPS
SNP effect on Acute myeloid leukaemia (controls excluding all cancers)
0.20
0.15
0.10
0.05
0.00
−0.05
0.05
0.06
0.07
0.08
SNP effect on Gut microbiota abundance (genus Lachnospiraceae ND3007 group id.11317)

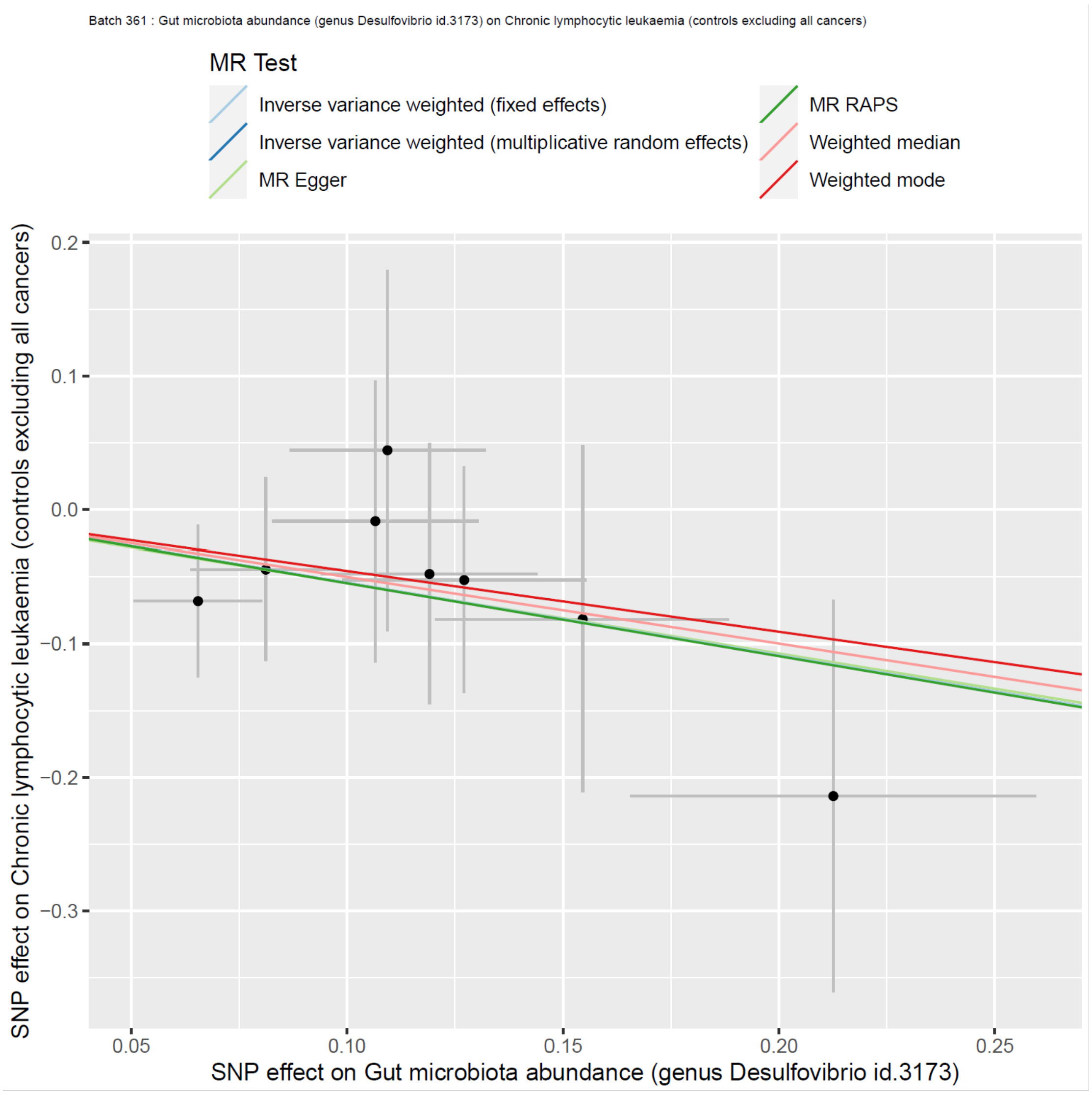

Batch 361 : Gut microbiota abundance (genus Desulfovibrio id.3173) on Chronic lymphocytic leukaemia (controls excluding all cancers)
MR Test
Inverse variance weighted (fixed effects)
Inverse variance weighted (multiplicative random effects)
MR Egger
MR RAPS
Weighted median
Weighted mode
0.2
0.1
0.0
−0.1
−0.2
−0.3
0.05
0.10
0.15
0.20
0.25
SNP effect on Chronic lymphocytic leukaemia (controls excluding all cancers)
SNP effect on Gut microbiota abundance (genus Desulfovibrio id.3173)

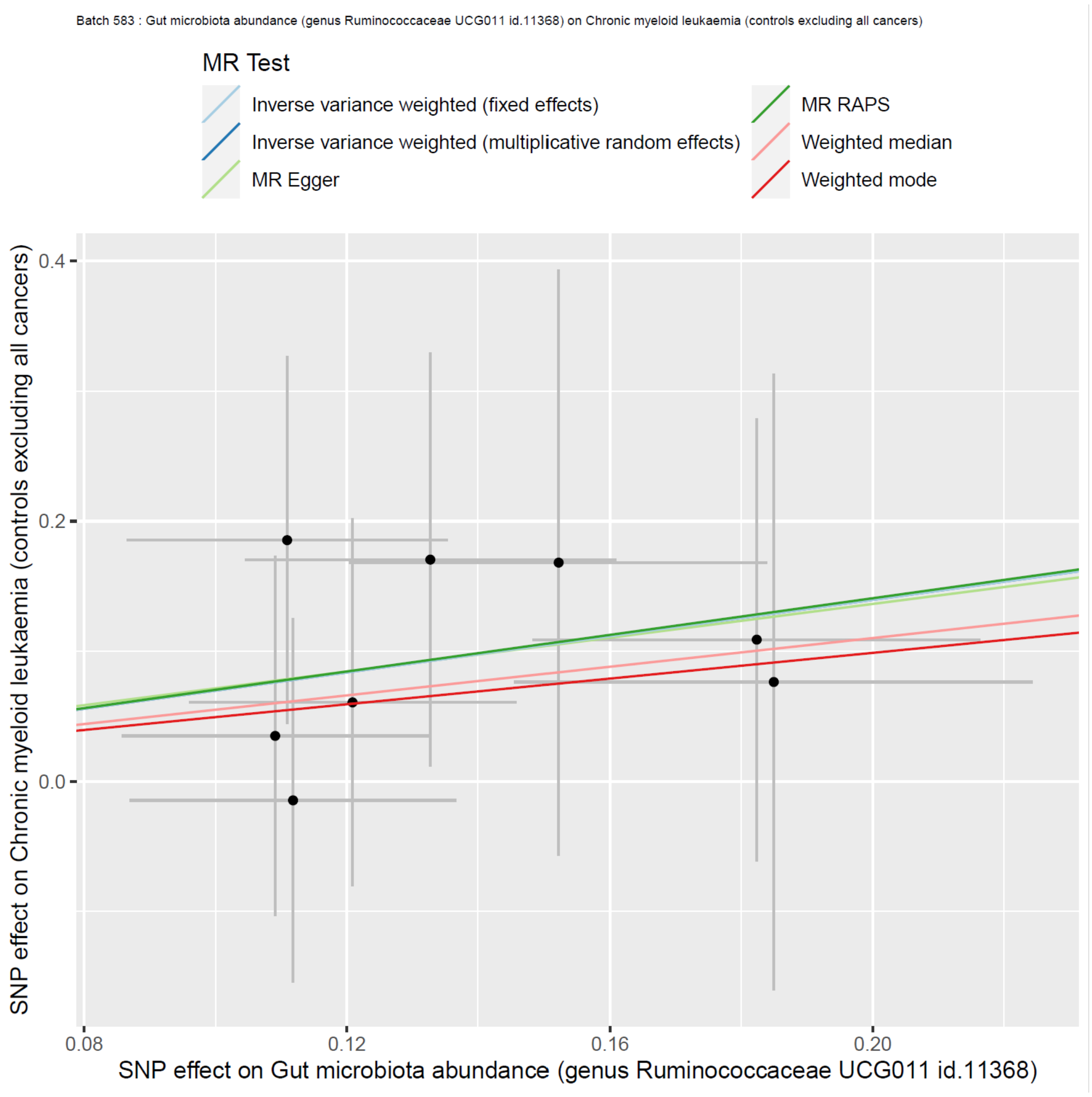

Batch 583 : Gut microbiota abundance (genus Ruminococcaceae UCG011 id.11368) on Chronic myeloid leukaemia (controls excluding all cancers)
MR Test
Inverse variance weighted (fixed effects)
Inverse variance weighted (multiplicative random effects)
MR Egger
MR RAPS
Weighted median
Weighted mode
SNP effect on Chronic myeloid leukaemia (controls excluding all cancers)
0.4
0.2
0.0
0.08
0.12
0.16
0.20
SNP effect on Gut microbiota abundance (genus Ruminococcaceae UCG011 id.11368)

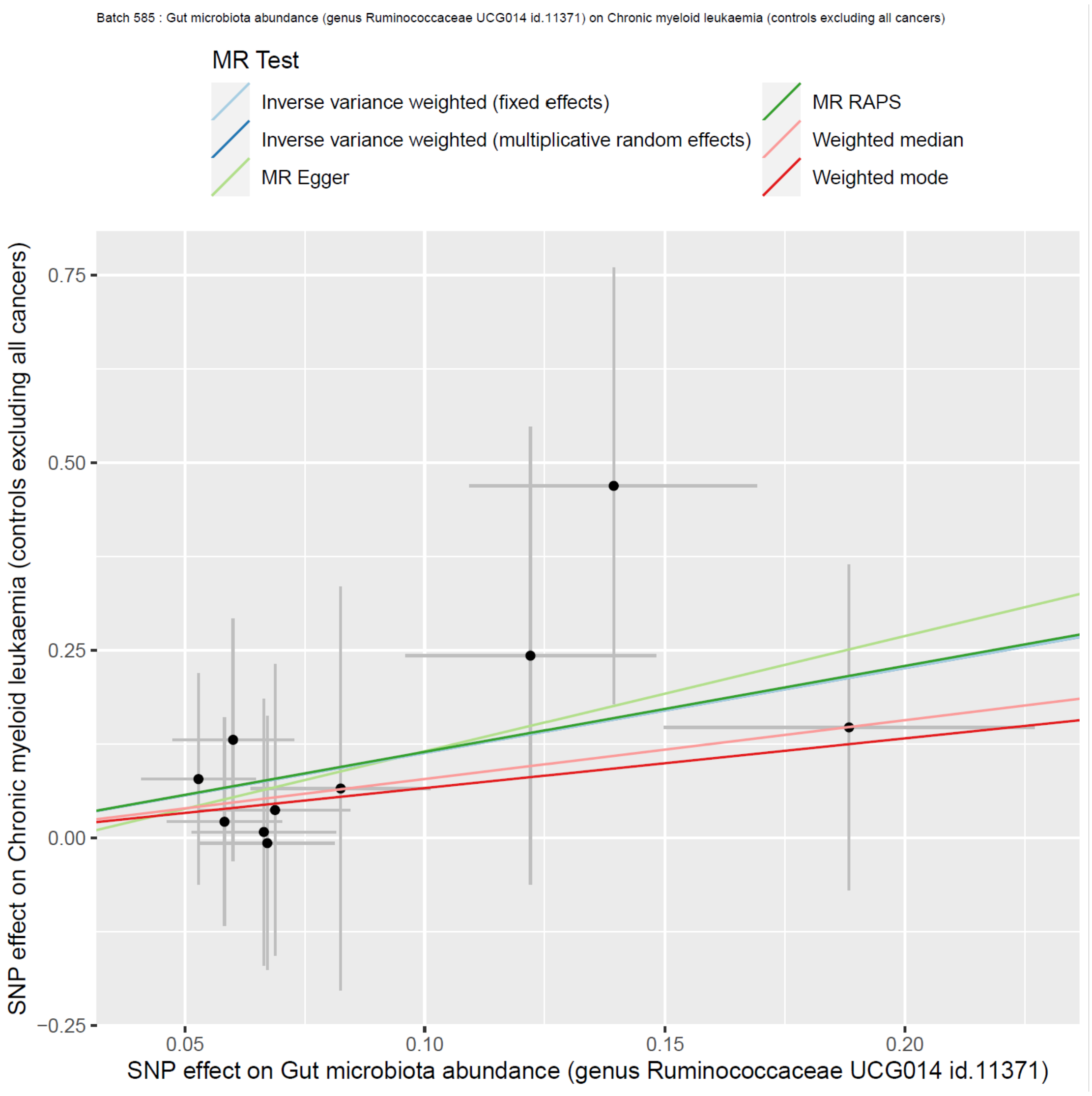

Batch 585 : Gut microbiota abundance (genus Ruminococcaceae UCG014 id.11371) on Chronic myeloid leukaemia (controls excluding all cancers)
MR Test
Inverse variance weighted (fixed effects)
Inverse variance weighted (multiplicative random effects)
MR Egger
MR RAPS
Weighted median
Weighted mode
SNP effect on Chronic myeloid leukaemia (controls excluding all cancers)
0.75
0.50
0.25
0.00
−0.25
0.05
0.10
0.15
0.20
SNP effect on Gut microbiota abundance (genus Ruminococcaceae UCG014 id.11371)
