# Supplementary material for: The causal relationship between gut microbiota and leukemia: a two-sample Mendelian randomization study
Source: Front Microbiol. 2023 Nov 22;14:1293333. doi: 10.3389/fmicb.2023.1293333 (PMC10703164; doi:10.3389/fmicb.2023.1293333)
Supplement: Supplementary file 4 [file Image_3.pdf]

Supplemental Figure 3."Leave-one-out" sensitivity analysis of causal effects of gut microbiota on leukemia.

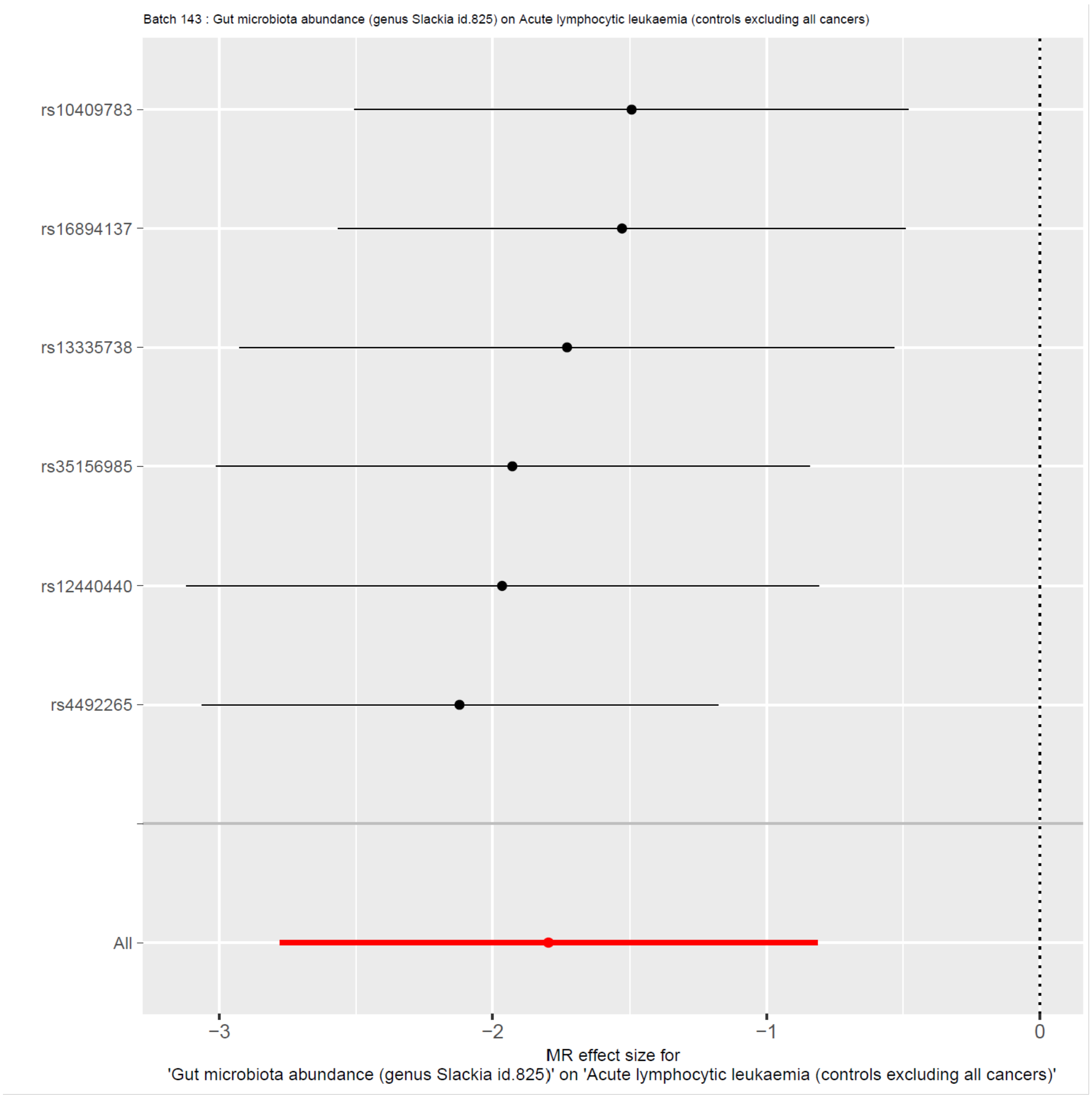

(A) Effect of genus Slackia on ALL

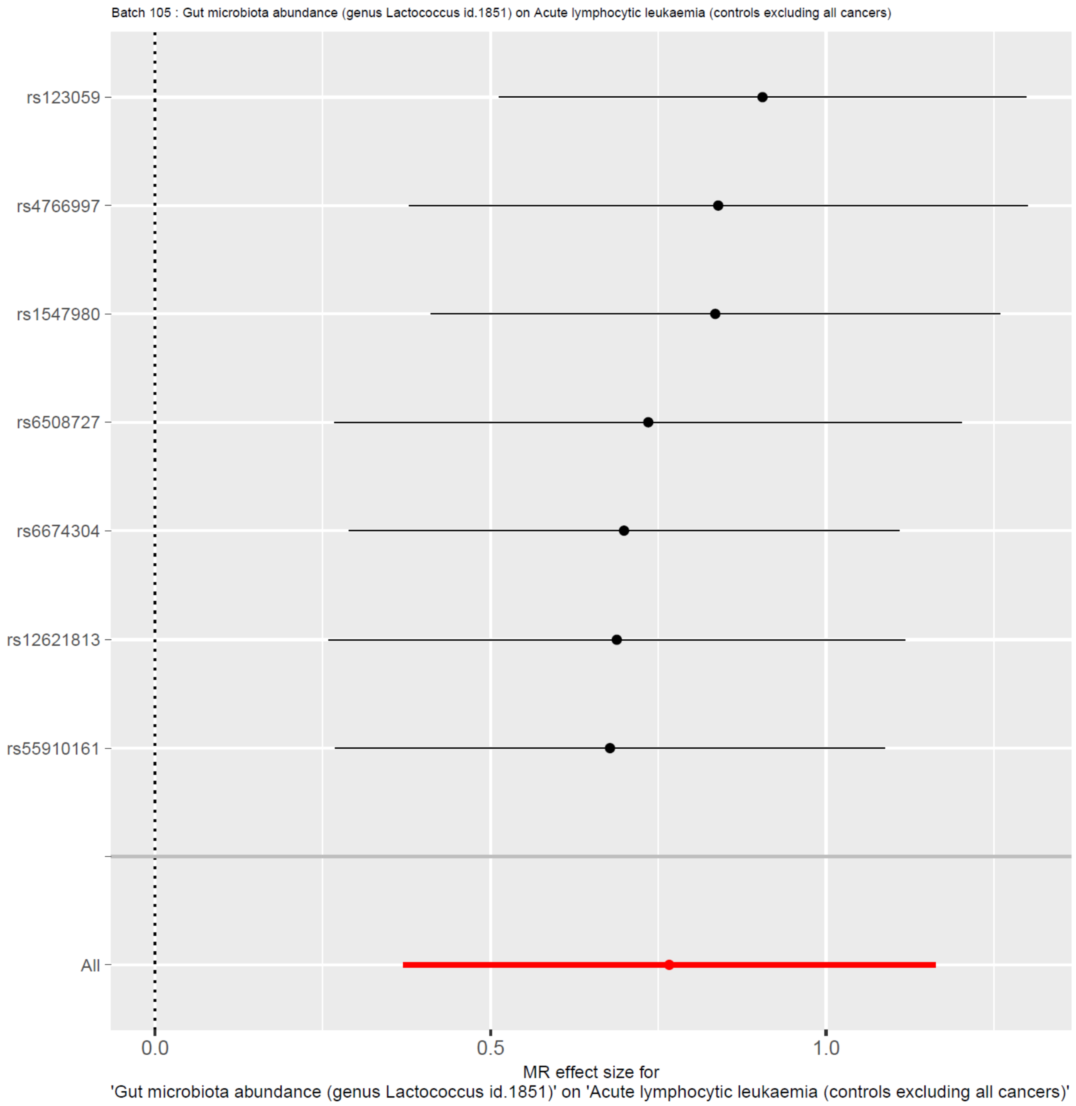

(B) Effect of genus Lactococcus on ALL

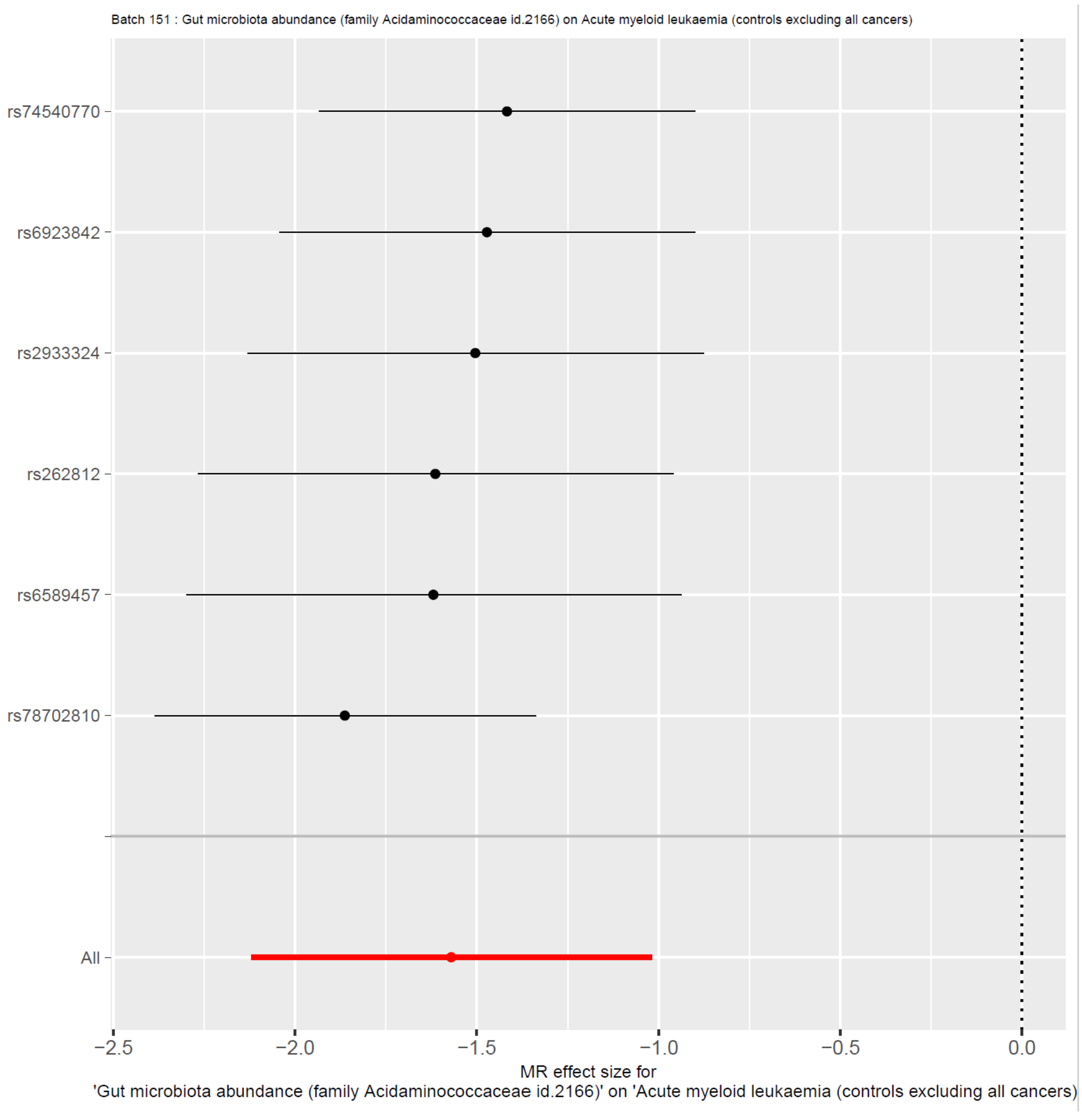

(C) Effect of family Acidaminococcaceae on AML

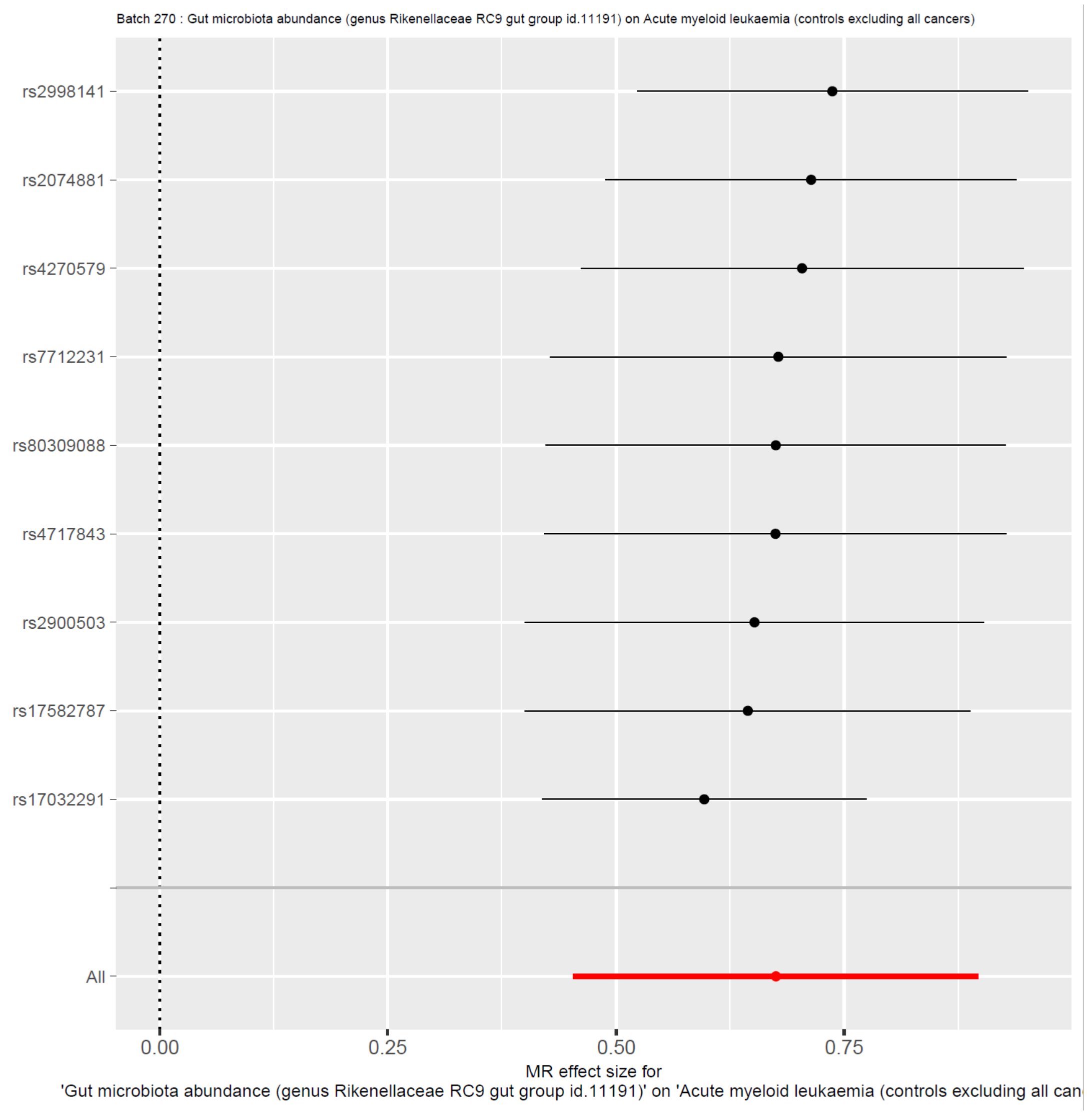

(D) Effect of genus Rikenellaceae RC9 gut group on AML

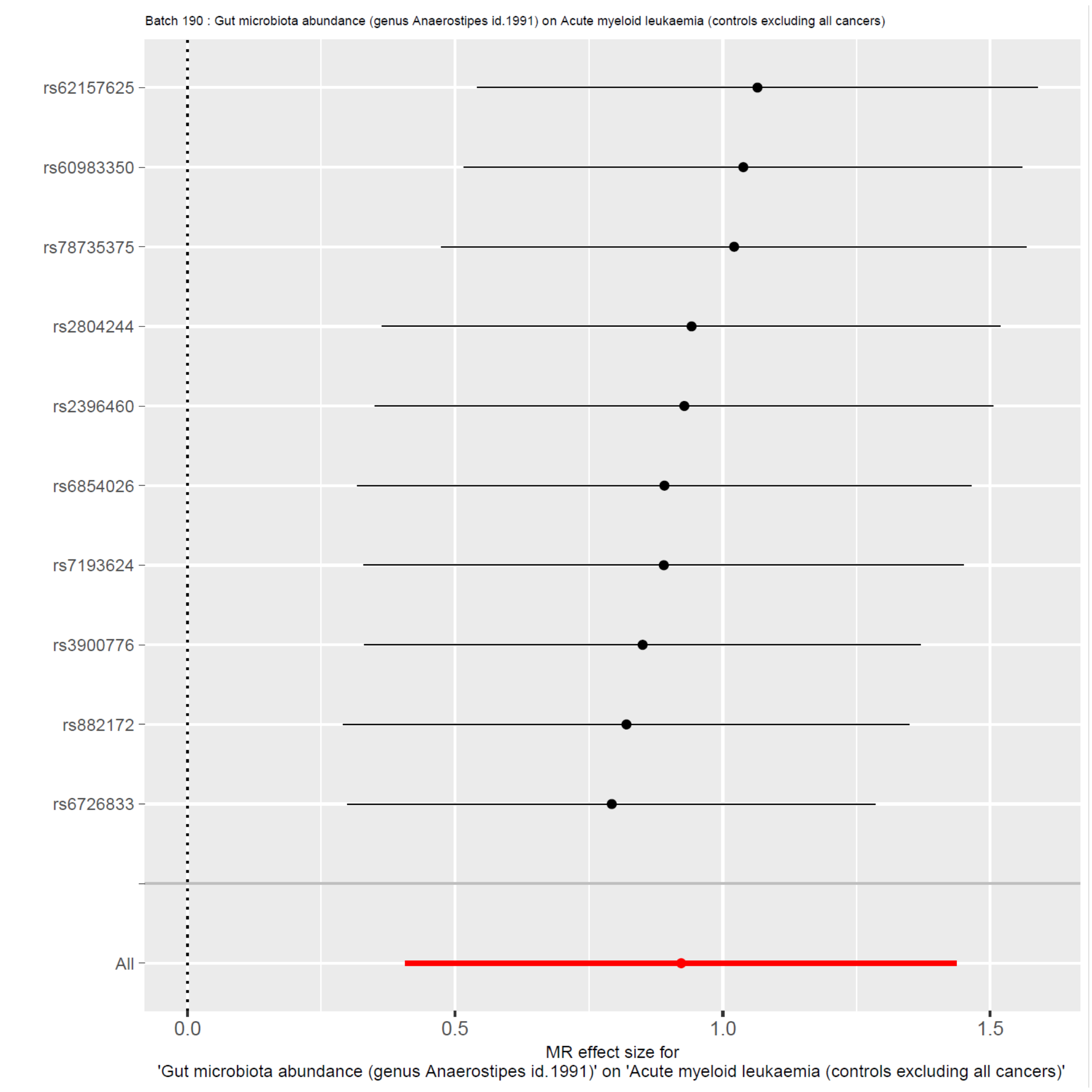

(E) Effect of genus Anaerostipes on AML

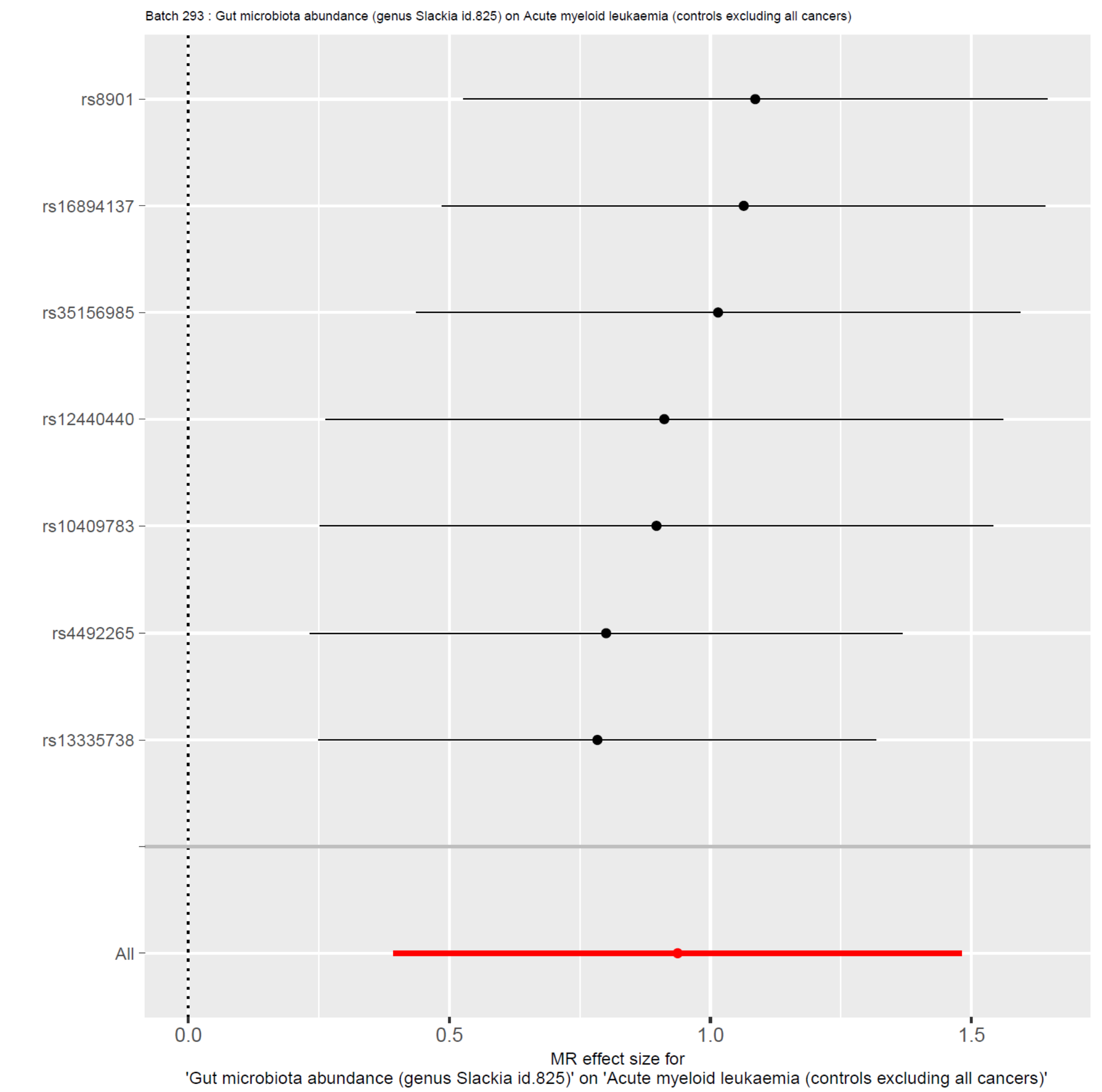

(F) Effect of genus Slackia on AML

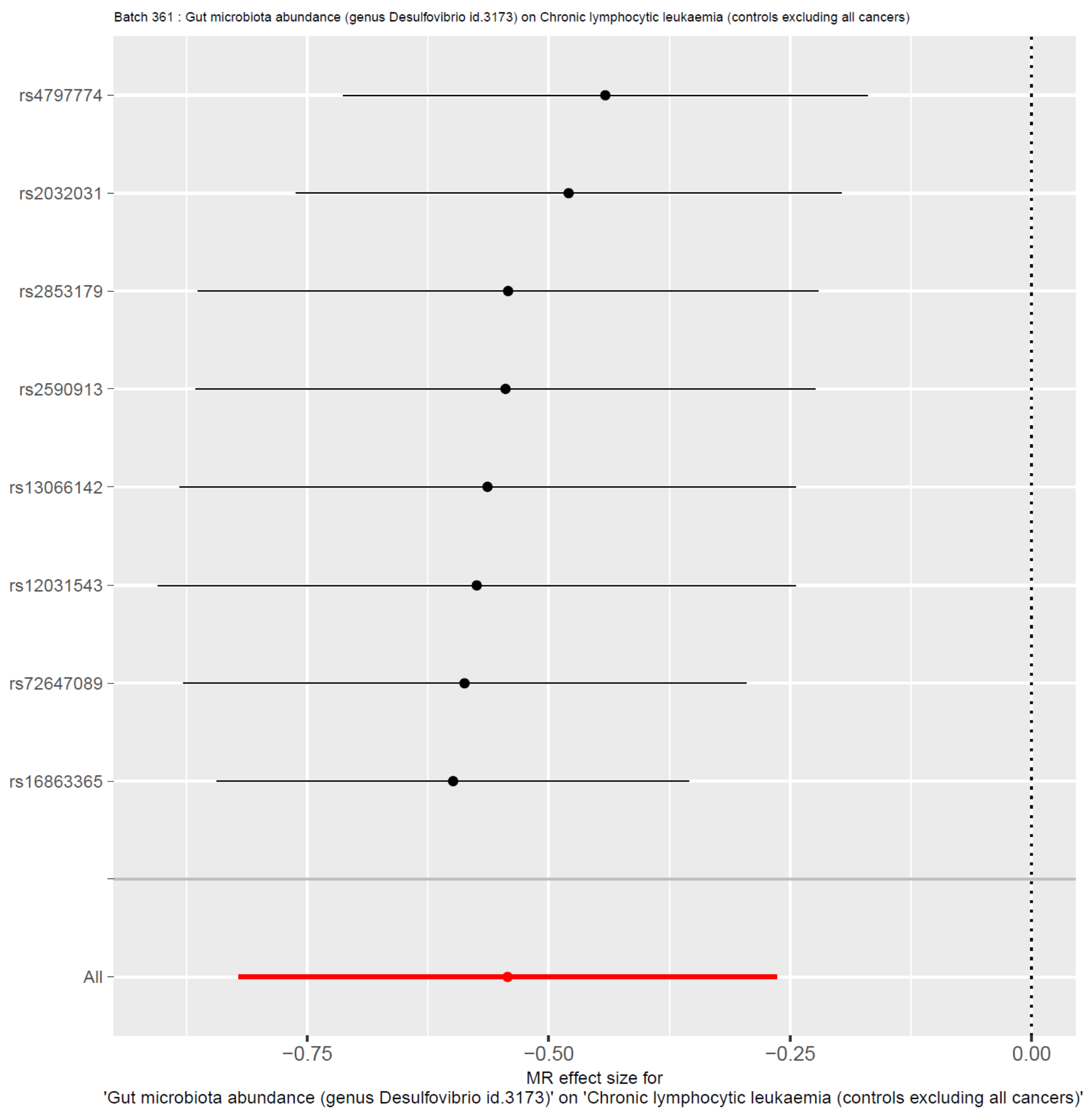

(G) Effect of genus Desulfovibrio on CLL

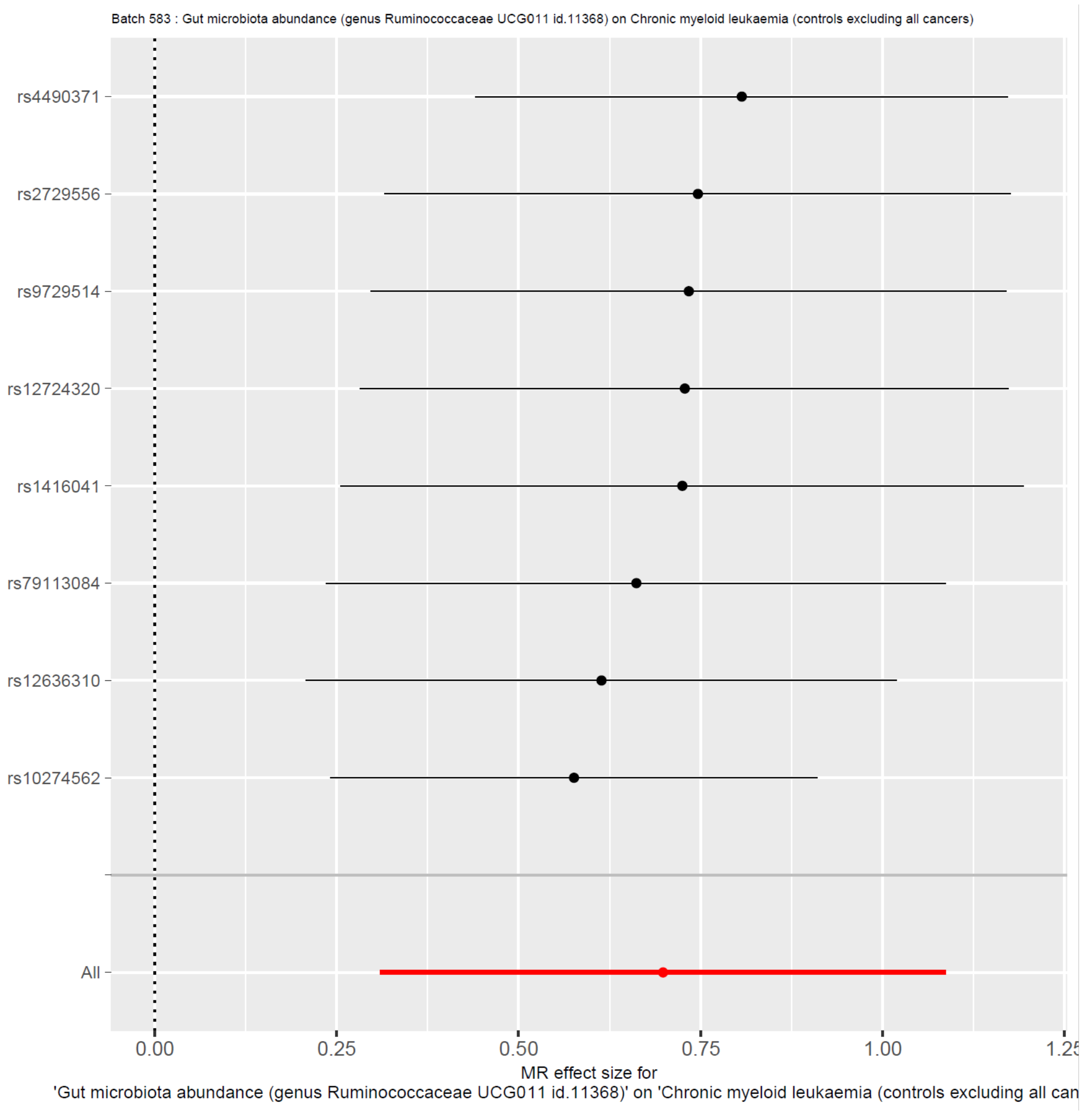

(H) Effect of genus Ruminococcaceae UCG011 on CML

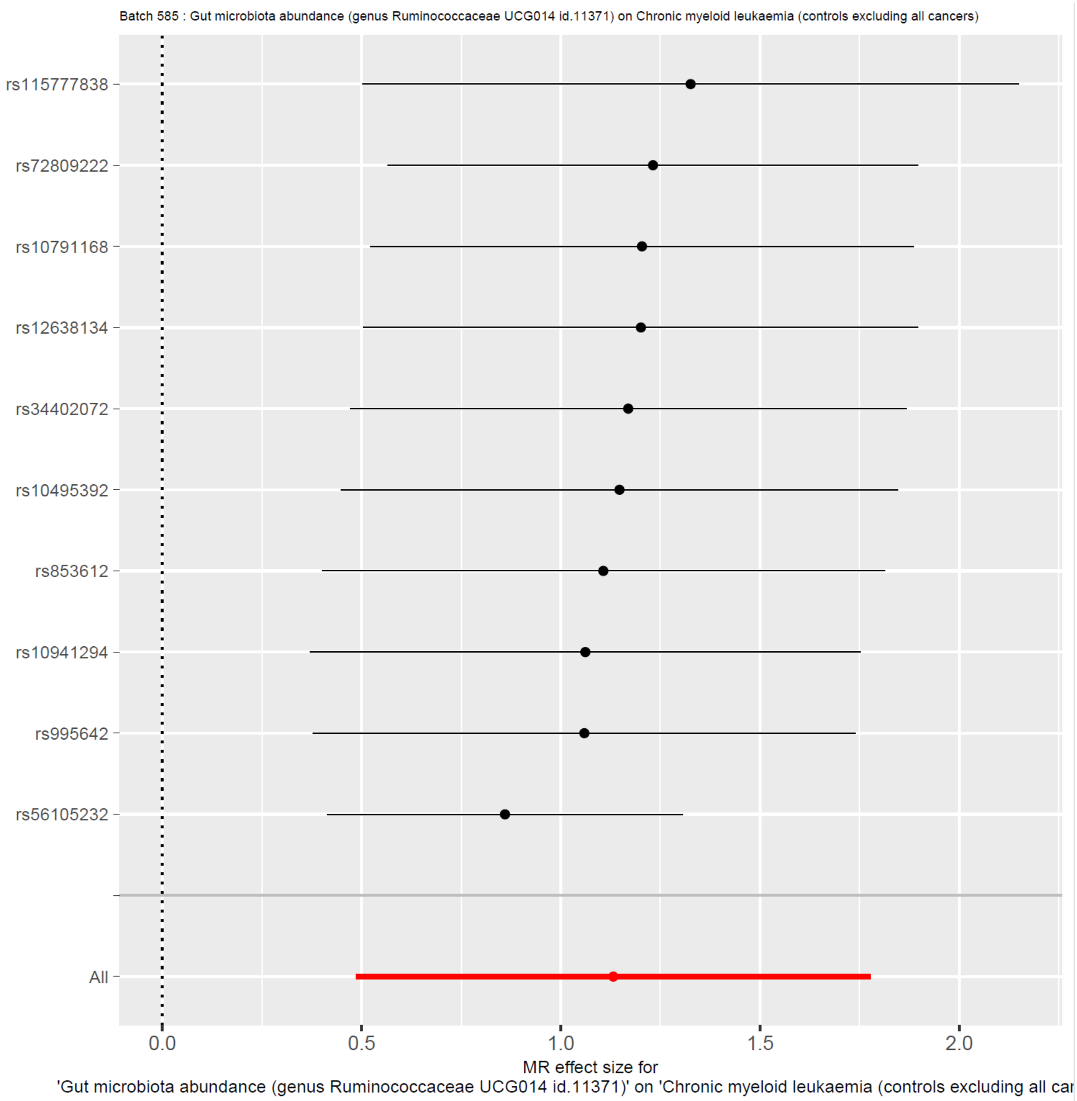

(I) Effect of genus Ruminococcaceae UCG014 on CML
