# Supplementary material for: The causal relationship between gut microbiota and leukemia: a two-sample Mendelian randomization study
Source: Front Microbiol. 2023 Nov 22;14:1293333. doi: 10.3389/fmicb.2023.1293333 (PMC10703164; doi:10.3389/fmicb.2023.1293333)
Supplement: Supplementary file 5 [file Image_4.pdf]

Supplementary Figure 4. Preliminary MR results and forest plots.

Exposures	NExp	Outcomes	NOut	NSNP	Method	OR (95% CI)	P value	Adjusted P value
genus Slackia	14306	Acute lymphocytic leukaemia (controls excluding all cancers)	287320	6	IVW(MRE)	0.166 (0.062 ~ 0.443)	<0.001	0.017
genus Christensenellaceae R 7group	14306	Acute lymphocytic leukaemia (controls excluding all cancers)	287320	8	IVW(MRE)	0.280 (0.096 ~ 0.819)	0.020	0.299
family Family XIII	14306	Acute lymphocytic leukaemia (controls excluding all cancers)	287320	7	IVW(MRE)	0.382 (0.174 ~ 0.842)	0.017	0.298
family Pasteurellaceae	14306	Acute lymphocytic leukaemia (controls excluding all cancers)	287320	13	IVW(MRE)	0.443 (0.244 ~ 0.802)	0.007	0.187
genus Odoribacter	14306	Acute lymphocytic leukaemia (controls excluding all cancers)	287320	7	IVW(MRE)	0.451 (0.233 ~ 0.872)	0.018	0.298
genus Terrisporobacter	14306	Acute lymphocytic leukaemia (controls excluding all cancers)	287320	4	IVW(MRE)	0.451 (0.239 ~ 0.852)	0.014	0.298
genus Ruminiclostridium6	14306	Acute lymphocytic leukaemia (controls excluding all cancers)	287320	15	IVW(MRE)	0.509 (0.270 ~ 0.960)	0.037	0.367
genus Blautia	14306	Acute lymphocytic leukaemia (controls excluding all cancers)	287320	2	IVW(MRE)	1.643 (1.592 ~ 1.695)	<0.001	<0.001
genus Catenibacterium	14306	Acute lymphocytic leukaemia (controls excluding all cancers)	287320	4	IVW(MRE)	1.663 (1.076 ~ 2.571)	0.022	0.300
family Family XI	14306	Acute lymphocytic leukaemia (controls excluding all cancers)	287320	8	IVW(MRE)	1.857 (1.053 ~ 3.277)	0.033	0.347
family Bifidobacteriaceae	14306	Acute lymphocytic leukaemia (controls excluding all cancers)	287320	9	IVW(MRE)	1.968 (1.074 ~ 3.607)	0.028	0.335
genus Lactococcus	14306	Acute lymphocytic leukaemia (controls excluding all cancers)	287320	7	IVW(MRE)	2.152 (1.447 ~ 3.199)	<0.001	0.011
genus Gordonibacter	14306	Acute lymphocytic leukaemia (controls excluding all cancers)	287320	8	IVW(MRE)	2.353 (1.090 ~ 5.080)	0.029	0.335
family Lachnospiraceae	14306	Acute lymphocytic leukaemia (controls excluding all cancers)	287320	14	IVW(MRE)	3.687 (1.482 ~ 9.172)	0.005	0.187
genus Dorea	14306	Acute lymphocytic leukaemia (controls excluding all cancers)	287320	10	IVW(MRE)	10.206 (1.858 ~ 56.055)	0.008	0.187
family Acidaminococcaceae	14306	Acute myeloid leukaemia (controls excluding all cancers)	287367	6	IVW(MRE)	0.208 (0.120 ~ 0.361)	<0.001	<0.001
genus Lachnospiraceae UCG010	14306	Acute myeloid leukaemia (controls excluding all cancers)	287367	9	IVW(MRE)	0.293 (0.110 ~ 0.785)	0.015	0.217
genus Faecalibacterium	14306	Acute myeloid leukaemia (controls excluding all cancers)	287367	10	IVW(MRE)	0.358 (0.164 ~ 0.779)	0.010	0.159
genus Subdoligranulum	14306	Acute myeloid leukaemia (controls excluding all cancers)	287367	8	IVW(MRE)	0.364 (0.173 ~ 0.765)	0.008	0.143
genus Defluviitaleaceae UCG011	14306	Acute myeloid leukaemia (controls excluding all cancers)	287367	9	IVW(MRE)	0.495 (0.249 ~ 0.983)	0.045	0.390
genus Parasutterella	14306	Acute myeloid leukaemia (controls excluding all cancers)	287367	14	IVW(MRE)	0.531 (0.296 ~ 0.954)	0.034	0.365
family Defluviitaleaceae	14306	Acute myeloid leukaemia (controls excluding all cancers)	287367	11	IVW(MRE)	0.563 (0.318 ~ 0.997)	0.049	0.390
genus Gordonibacter	14306	Acute myeloid leukaemia (controls excluding all cancers)	287367	7	IVW(MRE)	0.583 (0.340 ~ 0.999)	0.050	0.390
genus Eubacterium nodatum group	14306	Acute myeloid leukaemia (controls excluding all cancers)	287367	11	IVW(MRE)	0.644 (0.435 ~ 0.952)	0.027	0.314
family Oxalobacteraceae	14306	Acute myeloid leukaemia (controls excluding all cancers)	287367	13	IVW(MRE)	1.785 (1.097 ~ 2.906)	0.020	0.267
genus Erysipelatoclostridium	14306	Acute myeloid leukaemia (controls excluding all cancers)	287367	14	IVW(MRE)	1.817 (1.076 ~ 3.069)	0.025	0.314
genus Alloprevotella	14306	Acute myeloid leukaemia (controls excluding all cancers)	287367	5	IVW(MRE)	1.817 (1.204 ~ 2.744)	0.004	0.095
genus Rikenellaceae RC9 gut group	14306	Acute myeloid leukaemia (controls excluding all cancers)	287367	9	IVW(MRE)	1.964 (1.573 ~ 2.453)	<0.001	<0.001
genus Anaerostipes	14306	Acute myeloid leukaemia (controls excluding all cancers)	287367	10	IVW(MRE)	2.515 (1.503 ~ 4.209)	<0.001	0.017
genus Slackia	14306	Acute myeloid leukaemia (controls excluding all cancers)	287367	7	IVW(MRE)	2.553 (1.481 ~ 4.401)	0.001	0.022
genus Ruminiclostridium5	14306	Acute myeloid leukaemia (controls excluding all cancers)	287367	11	IVW(MRE)	2.631 (1.031 ~ 6.711)	0.043	0.390
genus Lachnospiraceae ND3007 group	14306	Acute myeloid leukaemia (controls excluding all cancers)	287367	2	IVW(MRE)	3.417 (1.960 ~ 5.959)	<0.001	0.001
family Pasteurellaceae	14306	Acute myeloid leukaemia (controls excluding all cancers)	287367	13	MR Egger #	5.253 (1.126 ~ 24.509)	0.037	0.369
genus Coprococcus1	14306	Acute myeloid leukaemia (controls excluding all cancers)	287367	9	IVW(MRE)	5.906 (1.778 ~ 19.620)	0.004	0.093
genus Lachnospiraceae ND3007 group	14306	Chronic lymphocytic leukaemia (controls excluding all cancers)	287757	2	IVW(MRE)	0.313 (0.124 ~ 0.791)	0.014	0.387
genus Desulfovibrio	14306	Chronic lymphocytic leukaemia (controls excluding all cancers)	287757	8	IVW(MRE)	0.581 (0.440 ~ 0.768)	<0.001	0.020
genus Ruminococcus gauvreauii group	14306	Chronic lymphocytic leukaemia (controls excluding all cancers)	287757	9	IVW(MRE)	0.582 (0.362 ~ 0.937)	0.026	0.402
genus Intestinimonas	14306	Chronic lymphocytic leukaemia (controls excluding all cancers)	287757	14	IVW(MRE)	0.632 (0.413 ~ 0.968)	0.035	0.431
genus Eubacterium brachy group	14306	Chronic lymphocytic leukaemia (controls excluding all cancers)	287757	10	IVW(MRE)	0.673 (0.474 ~ 0.956)	0.027	0.402
genus Turicibacter	14306	Chronic lymphocytic leukaemia (controls excluding all cancers)	287757	9	IVW(MRE)	0.710 (0.529 ~ 0.952)	0.022	0.402
genus Allisonella	14306	Chronic lymphocytic leukaemia (controls excluding all cancers)	287757	7	IVW(MRE)	0.767 (0.619 ~ 0.950)	0.015	0.387
family Methanobacteriaceae	14306	Chronic lymphocytic leukaemia (controls excluding all cancers)	287757	9	IVW(MRE)	1.383 (1.063 ~ 1.798)	0.016	0.387
genus Howardella	14306	Chronic lymphocytic leukaemia (controls excluding all cancers)	287757	9	IVW(MRE)	1.445 (1.139 ~ 1.834)	0.002	0.181
genus Lactococcus	14306	Chronic lymphocytic leukaemia (controls excluding all cancers)	287757	7	IVW(MRE)	1.479 (1.016 ~ 2.153)	0.041	0.472
genus Ruminococcaceae UCG010	14306	Chronic lymphocytic leukaemia (controls excluding all cancers)	287757	6	IVW(MRE)	1.481 (1.048 ~ 2.092)	0.026	0.402
genus Flavonifractor	14306	Chronic lymphocytic leukaemia (controls excluding all cancers)	287757	5	IVW(MRE)	2.113 (1.060 ~ 4.211)	0.033	0.431
family Oxalobacteraceae	14306	Chronic lymphocytic leukaemia (controls excluding all cancers)	287757	13	MR Egger #	8.085 (2.026 ~ 32.270)	0.007	0.337
genus Erysipelatoclostridium	14306	Chronic myeloid leukaemia (controls excluding all cancers)	287242	14	MR Egger #	0.006 (0.000 ~ 0.500)	0.027	0.509
family Veillonellaceae	14306	Chronic myeloid leukaemia (controls excluding all cancers)	287242	18	MR Egger #	0.067 (0.006 ~ 0.712)	0.027	0.509
genus Eubacterium fissicatena group	14306	Chronic myeloid leukaemia (controls excluding all cancers)	287242	8	IVW(MRE)	0.384 (0.158 ~ 0.930)	0.034	0.557
genus Defluviitaleaceae UCG011	14306	Chronic myeloid leukaemia (controls excluding all cancers)	287242	9	IVW(MRE)	1.930 (1.010 ~ 3.690)	0.047	0.665
genus Ruminococcaceae UCG011	14306	Chronic myeloid leukaemia (controls excluding all cancers)	287242	8	IVW(MRE)	2.010 (1.363 ~ 2.963)	<0.001	0.044
genus Ruminococcaceae UCG005	14306	Chronic myeloid leukaemia (controls excluding all cancers)	287242	14	IVW(MRE)	2.979 (1.399 ~ 6.342)	0.005	0.171
genus Eggerthella	14306	Chronic myeloid leukaemia (controls excluding all cancers)	287242	9	IVW(MRE)	3.015 (1.178 ~ 7.719)	0.021	0.509
genus Ruminococcaceae UCG014	14306	Chronic myeloid leukaemia (controls excluding all cancers)	287242	10	IVW(MRE)	3.101 (1.626 ~ 5.915)	0.001	0.044
genus Flavonifractor	14306	Chronic myeloid leukaemia (controls excluding all cancers)	287242	5	IVW(MRE)	4.039 (1.629 ~ 10.016)	0.003	0.128
genus Ruminococcaceae UCG002	14306	Chronic myeloid leukaemia (controls excluding all cancers)	287242	16	IVW(MRE)	5.221 (1.494 ~ 18.244)	0.010	0.285

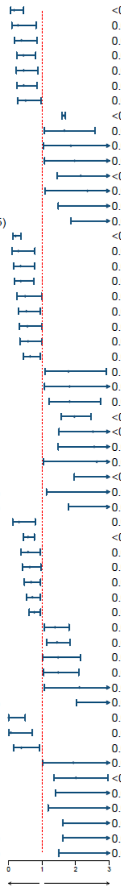

* refer to existence of heterogeneity between SNPs, # refer to existence of pleiotropy of SNPs
